# Effects of different intercropping patterns on tea rhizosphere environment and leaf quality

**DOI:** 10.3389/fpls.2026.1914455

**Published:** 2026-08-11

**Authors:** Xiaohua Wang, Wenxia Yuan, Weihao Liu, Miao Zhou, Junjie He, Guangxing Li, Zihua Zhao, Xinghua Wang, Zuzhen Wang, Qiaomei Wang, Baijuan Wang

**Affiliations:** 1 College of Tea Science, Yunnan Agricultural University, Kunming, China; 2 Yunnan Organic Tea Industry Intelligent Engineering Research Center, Kunming, China; 3 College of Agronomy and Biotechnology, Yunnan Agricultural University, Kunming, China; 4 Comprehensive Support and Technical Service Center, Wongdu Town, Changning County, Baoshan, Yunnan, China

**Keywords:** high-throughput sequencing, rhizosphere microbial community, soil physicochemical properties, tea plantation intercropping, tea quality

## Abstract

Intercropping can improve tea soils and leaf quality, but partner-specific responses remain unclear. We compared four systems—*Paris polyphylla*–tea (CT), leguminous green manure–tea (LF), *Pleurotus ostreatus*–tea (CJ), and *Polygonatum kingianum*–tea (HJ)—with matched monoculture controls. Tea and soil traits were integrated with high-throughput sequencing, LEfSe, PICRUSt2 functional prediction, and correlation analyses. LF significantly increased soil available and total phosphorus, enriched the potentially beneficial fungi *Mortierella* and *Trichoderma* in the tea rhizosphere, and enhanced the predicted potential for microbial amino acid transformation, potentially facilitating leaf free amino acid accumulation, which increased by 28%. CT enriched *Penicillium* and enhanced predicted microbial carbohydrate-conversion and redox potential, potentially promoting leaf flavonoid accumulation, which increased by 21%. CJ significantly increased soil available phosphorus and potassium, enriched *Talaromyces* and *Exophiala*, and enhanced predicted organic-substrate-transformation potential, potentially facilitating organic matter turnover and nutrient release. HJ increased soil pH by 24%, enriched Nitrosomonadaceae and Rhizobiaceae, and enhanced predicted amino acid-, carbohydrate-, and phenylpropanoid-related metabolic potential, indicating improved acid-soil conditions and distinct microbial functional responses. Overall, different partners improved different attributes. LF showed the most balanced contribution to soil phosphorus status and amino-acid-associated tea quality, whereas CT, CJ, and HJ offered targeted advantages in flavonoid enrichment, phosphorus and potassium availability, and acid-soil amelioration, respectively. These findings support selecting intercropping partners according to specific soil-management and tea-quality objectives.

## 1 Introduction

Tea, produced from the young shoots of *Camellia sinensis*, is an economically important perennial crop whose sensory attributes and health-related properties are closely associated with its free amino acids, tea polyphenols, soluble sugars, and flavonoids (Yu et al., 2020). The accumulation of these compounds is regulated not only by plant genotype and climatic conditions but also by management-induced changes in the root-zone environment. By introducing partner-specific root-derived inputs, plant residues, and resource-use patterns, intercropping can alter soil physicochemical properties, nutrient availability, enzyme activities, and rhizosphere bacterial and fungal communities. These belowground changes may subsequently influence carbon and nitrogen acquisition and allocation by tea plants, resulting in differential accumulation of tea quality-related compounds (Chen et al., 2025; Ma et al., 2017; Sun et al., 2022).

Evidence from tea-based intercropping systems supports this soil–microbiome–plant linkage. Legume intercropping and green-manure incorporation can introduce biologically fixed nitrogen and organic matter, modify soil pH and enzyme activities, and restructure microbial communities involved in nutrient mineralization and turnover. These changes have been associated with corresponding variation in tea-leaf amino acids, soluble sugars, polyphenols, and other quality-related components (Duan et al., 2024; Wang et al., 2023; Zhou et al., 2024). Metabolomic evidence further indicates that legume intercropping can regulate amino acid, carbohydrate, and flavonoid metabolic pathways in tea plants rather than simply increasing the total soil nutrient pool (Duan et al., 2021). Soil pH is another important regulator because it affects elemental solubility, root nutrient uptake, and tea-leaf biochemical quality (Jia et al., 2024). Thus, intercropping outcomes depend jointly on intercrop identity, residue management, nutrient inputs, rhizosphere microbial recruitment, and microbial nutrient turnover, which determine whether nutrients are mineralized, retained, or temporarily immobilized. Edible-fungus cultivation represents a contrasting pathway: tea–*Pleurotus ostreatus* systems introduce lignocellulosic substrates and fungal mycelia, modify decomposer communities and soil nutrient pools, and influence tea-leaf secondary metabolites (Ma et al., 2022; Yang et al., 2024). Intercropping effects are therefore expected to be partner-specific rather than uniformly beneficial.

The four intercropping partners examined in this study were selected to represent contrasting ecological functions and production uses. *Vicia villosa* is a leguminous green manure whose symbiotic nitrogen fixation and subsequent biomass incorporation can supply labile carbon and nitrogen and stimulate microbial nutrient turnover, making it particularly relevant to nitrogen-dependent amino acid formation in tea leaves (Huang et al., 2022). *Pleurotus ostreatus* is an edible fungus suitable for cultivation within tea interrows; its cultivation substrate and postharvest residues provide organic inputs for decomposer microorganisms and nutrient cycling while generating an additional economic product. *Paris polyphylla* and *Polygonatum kingianum* are perennial medicinal plants with potential value in diversified agroforestry systems in southwestern China. Intercropping *Paris polyphylla* in a woody perennial system has been shown to modify rhizosphere pH, nutrient availability, and bacterial and fungal community composition (Zhang et al., 2019). The growth and medicinal quality of *Polygonatum kingianum* are likewise closely associated with soil nutrient conditions and rhizosphere microbial composition (Liu et al., 2024). Collectively, these four partners provide a functional contrast among nitrogen-fixing green manure, fungus-mediated organic-substrate decomposition, and two medicinal-plant rhizosphere systems.

Despite increasing interest in tea intercropping, previous studies have generally evaluated a single intercropping partner or compared species belonging to the same functional category, often under different environmental and management conditions. Comparisons among such studies cannot reliably separate partner-specific effects from differences caused by site conditions, tea cultivars, residue management, or fertilization. Moreover, soil physicochemical properties, bacterial and fungal communities, discriminatory microbial taxa, predicted functional potential, and tea biochemical quality have rarely been evaluated together within the same experimental framework. Consequently, it remains unclear why one intercropping configuration may favor amino acid accumulation, whereas another primarily affects polyphenols, soluble sugars, or flavonoids. A same-site comparison of functionally contrasting intercropping partners is therefore required to identify configuration-specific benefits and trade-offs and to develop testable soil–microbiome–tea quality pathways.

Accordingly, this study compared four intercropping configurations—tea with *Paris polyphylla* (CT), Leguminous green manure (LF), *Pleurotus ostreatus* (CJ), or *Polygonatum kingianum* (HJ)—with their corresponding independent monoculture tea controls. The objectives were to: (i) quantify the effects of intercropping configuration, cropping system, and their interaction on tea biochemical and soil physicochemical properties; (ii) characterize changes in rhizosphere bacterial and fungal diversity and community composition and identify discriminatory taxa; (iii) evaluate configuration-specific shifts in predicted microbial functional potential; and (iv) examine the associations among soil properties, rhizosphere microbial communities, and tea biochemical traits. We hypothesized that the contrasting functional traits and organic inputs of the four intercropping partners would generate distinct soil and microbial responses and, consequently, differential effects on tea quality components.

## 2 Materials and methods

### 2.1 Environmental conditions and field experimental layout

The field experiment was conducted in Tujie Town, Nanhua County, Chuxiong Yi Autonomous Prefecture, Yunnan Province, China (101°51′38.01″ E, 25°20′4.18″ N). The region has a subtropical monsoon climate, with a mean annual temperature of 15.6 °C and annual precipitation of approximately 850 mm. The soil at the experimental site is classified as red soil according to the Chinese soil classification system.

Tea plants were arranged in rows spaced 1.5 m apart, with a within-row plant spacing of 0.4 m. Four intercropping configurations and their corresponding independent monoculture tea controls were established in March 2020. The spatial arrangements were as follows:

1. *Paris polyphylla—*tea (CT): *Paris polyphylla* was planted in parallel rows between adjacent tea rows, with both row-to-row and plant-to-plant spacings of 0.5 m.
2. Leguminous green manure—tea (LF): *Vicia villosa* Roth (hairy vetch) was uniformly broadcast between the tea rows at an approximate seeding rate of 30–45 kg ha^−1^. Approximately 20 t ha^−1^ of fresh biomass was incorporated into the soil at peak flowering.
3. *Pleurotus ostreatus—*tea (CJ): Shade shelters were constructed over the *Pleurotus ostreatus* cultivation areas between adjacent tea rows without covering or shading the tea canopy. Cultivation trenches, approximately 20 cm wide and 10 cm deep, were established 10–15 cm from the tea plants. The spent mushroom substrate was incorporated into the soil in autumn.
4. *Polygonatum kingianum—*tea (HJ): *Polygonatum kingianum* was planted in parallel rows between adjacent tea rows, with a row spacing of 0.40 m and a within-row plant spacing of 0.30–0.40 m.

Each treatment comprised three independent replicate plots, each covering 20 m² (5 m × 4 m). The corresponding monoculture controls (CTC, LFC, CJC, and HJC) had the same tea planting density and field-management practices as their respective intercropping treatments but contained no intercrops. Throughout the experimental period, all plots received consistent field management, including routine weeding and environmentally friendly pest control, without the application of chemical fertilizers or pesticides. The CJ treatment were restricted to the mushroom cultivation areas between the tea rows and did not cover or directly shade the tea canopy. Thus, the tea plants remained exposed to ambient field light.

### 2.2 Collection and processing of samples

Samples were collected on April 16, 2024, during the spring tea harvesting period. Five sampling points were selected within each plot. Healthy tea plants without visible pest or disease symptoms were selected, and fine roots with closely adhering rhizosphere soil were collected within a 20-cm radius around the base of each selected tea plant to a depth of 30 cm (Lin et al., 2019). Soil obtained from the five sampling points within each plot was combined to form one composite biological replicate. Each treatment therefore comprised three independent composite samples (n = 3). The samples were placed in sterile resealable bags, transported to the laboratory at 4 °C, and processed immediately.

Each composite soil sample was divided into two subsamples. One subsample was air-dried, passed through a 2-mm sieve, and used for physicochemical analyses; the other was immediately stored at −80 °C until microbial DNA extraction. Meanwhile, fresh tea shoots consisting of one apical bud and the first two leaves were collected from the same plots. The shoots were immediately fixed by pan-firing to inactivate endogenous enzymes, dried at 60 °C for 4 h, ground into a homogeneous powder, passed through an 80-mesh sieve, and stored in airtight bags until biochemical analysis.

### 2.3 Measurement indices and methods

#### 2.3.1 Determination of soil physicochemical parameters and nutrient status

All soil physicochemical properties were analyzed using established soil chemical procedures adapted from Sparks et al. (1996), as detailed below. Soil acidity pH was assessed potentiometrically in soil-water suspension. The soil organic matter (SOM) fraction was determined using the thermally-assisted potassium dichromate oxidation method. For the assessment of elemental nitrogen status, the Kjeldahl digestion protocol was employed to quantify total nitrogen (TN) concentrations. The levels of both total and available phosphorus (TP and AP) were evaluated through molybdenum-antimony colorimetry, whereas total and available potassium (TK and AK) concentrations were acquired using a flame photometer. Finally, the ammonium acetate exchange procedure was utilized to determine the cation exchange capacity (CEC).

#### 2.3.2 Determination of tea quality components

To quantify the biochemical metabolites associated with tea quality, a series of standardized colorimetric assays were conducted. The concentration of tea polyphenols was evaluated using the Folin–Ciocalteu method, strictly adhering to the Chinese National Standard GB/T 8313–2018. For the assessment of flavonoids, the aluminum chloride protocol described by Ordóñez et al. (2006) was applied. Furthermore, the anthrone–sulfuric acid procedure served to quantify soluble sugars, while the total free amino acid profile was determined via ninhydrin colorimetry in accordance with the GB/T 8314–2013 guidelines (Standardization Administration of China, 2013).

#### 2.3.3 Analysis of microbial community structure

Soil metagenomic DNA was isolated from the specimens, with its integrity subsequently confirmed via 1% agarose gel electrophoresis. To profile the bacterial and fungal microbiomes, specific barcode-tagged primer sets were utilized for targeted amplification. The bacterial 16S rRNA gene (V3–V4 region) subregions were enriched using the 338F/806R primer pair (5′-ACTCCTACGGGAGGCAGCAG-3′ and 5′-GGACTACHVGGGTWTCTAAT-3′), whereas the fungal internal transcribed spacer (ITS) segments were captured with the ITS1F/ITS4R primers (5’-CTTGGTCATTTAGAGGAAGTAA-3’ and 5’-TCCTCCGCTTATTGATATGC-3’).

Each amplification reaction was set up in a 20 μL mixture containing 10 ng of DNA, 4 μL of 5× TransStart FastPfu buffer, 2 μL of 2.5 mM dNTPs, 0.4 μL of TransStart FastPfu DNA polymerase, and 0.8 μL of each primer (5 μM), with nuclease-free water added to complete the volume. Thermocycling was carried out using an ABI GeneAmp^®^ 9700 (USA) and started with an initial denaturation step at 95 °C for 3 minutes. The reaction then proceeded with 27 cycles of 30 seconds of denaturation at 95 °C, 30 seconds of annealing (55 °C for bacterial 16S, 60 °C for fungal ITS), and a 45-second extension at 72 °C. A final extension at 72 °C for 10 minutes was followed by cooling at 4 °C.

Target amplicons were resolved on an agarose-based gel (2% concentration), subsequently recovered, and purified using a specialized PCR Clean-Up Kit (Yuhua, China). The concentration of the recovered DNA was fluorometrically determined via a Qubit 4.0 system. Sequencing libraries were generated strictly adhering to the NEXTFLEX^®^ Rapid DNA-Seq Kit guidelines. The preparation workflow encompassed adapter ligation, the elimination of self-ligated artifacts via bead-based size selection, template enrichment through PCR, and a terminal magnetic bead clean-up step. Finally, high-throughput sequencing of the constructed libraries was executed on an Illumina NextSeq 2000 platform, facilitated by Majorbio Bio-Pharm Technology Co., Ltd. (Shanghai, China).

### 2.4 Data analysis

Raw sequencing reads were processed using QIIME 2 (v2022.11). Amplicon sequence variants (ASVs) were generated using the DADA2 pipeline after quality filtering, denoising, merging, and chimera removal. Mitochondrial and chloroplast sequences were excluded, and the sequencing depth was rarefied to 6,000 reads per sample before downstream analyses. Bacterial and fungal ASVs were taxonomically assigned using the SILVA v138 and UNITE v8.0 reference databases, respectively.

The Shannon and Chao indices were calculated using Mothur (v1.48.0) to characterize microbial α-diversity. Overall differences among the eight treatment groups were evaluated using the Kruskal–Wallis H test. When the overall test was significant, Dunn’s *post-hoc* test with Bonferroni correction was used for pairwise comparisons. Principal coordinate analysis (PCoA) was conducted using Bray–Curtis dissimilarities, and differences in microbial community composition among treatments were evaluated using permutational multivariate analysis of variance (PERMANOVA), with statistical significance determined at P < 0.05. Differential microbial taxa were identified using linear discriminant analysis effect size (LEfSe), with an LDA score ≥ 3.0 and P < 0.05. Fungal biomarkers were visualized at the genus level, whereas bacterial biomarkers were visualized at the family level because a substantial proportion of bacterial ASVs could not be confidently classified at the genus level.

Marker-gene-based functional inference was performed separately for the bacterial 16S rRNA gene and fungal ITS datasets using PICRUSt2 v2.2.0 through the Majorbio Cloud Platform workflow (v2.2.0-b; Douglas et al., 2020). The corresponding ASV abundance tables and taxonomic annotations were used as input. Predicted functional profiles were summarized using KEGG Orthology, Enzyme Commission (EC), and COG categories. Differentially represented predicted enzymes were compared between each intercropping treatment and its corresponding monoculture control. Because these profiles were inferred from marker-gene data rather than directly measured using metagenomics, metatranscriptomics, or enzyme-activity assays, they were interpreted as predicted functional potentials rather than actual microbial metabolic activities.

Tea biochemical and soil physicochemical parameters were analyzed separately using two-way analysis of variance (ANOVA), with intercropping configuration (CT, LF, CJ, and HJ) and cropping system (intercropping and corresponding monoculture) as fixed factors. Their main effects and interactions were included in the statistical model. The normality of model residuals and homogeneity of variance were evaluated using the Shapiro–Wilk and Levene tests, respectively. When significant effects were detected, Tukey’s honestly significant difference (HSD) test was used for multiple comparisons among the eight treatment combinations. Statistical significance was determined at P < 0.05. Data are presented as the mean ± standard deviation (SD) from three independent biological replicates (n = 3). These analyses were performed using IBM SPSS Statistics v26.0.

Pearson correlation analysis was used to evaluate pairwise associations between soil physicochemical properties and tea biochemical traits. For the integrated Mantel analysis, environmental and tea-quality variables were standardized before distance calculation. Bray–Curtis dissimilarity matrices were constructed for the microbial community profiles and the standardized factor data. Mantel correlations between the distance matrices were calculated using Pearson’s correlation coefficient, and statistical significance was evaluated using 999 permutations. Relationships with P < 0.05 were considered statistically significant. The heatmap reports Pearson’s r for correlations among individual physicochemical and biochemical variables, whereas the connecting lines report Mantel’s r and the corresponding permutation-based Mantel P-values for associations with bacterial or fungal community composition.

The bioinformatic and integrated multivariate analyses were conducted using the Majorbio Cloud Platform and R v4.2.2, and the figures were prepared using Origin 2024.

## 3 Results

### 3.1 Effects of different intercropping systems on tea quality components

**Figure 1:**
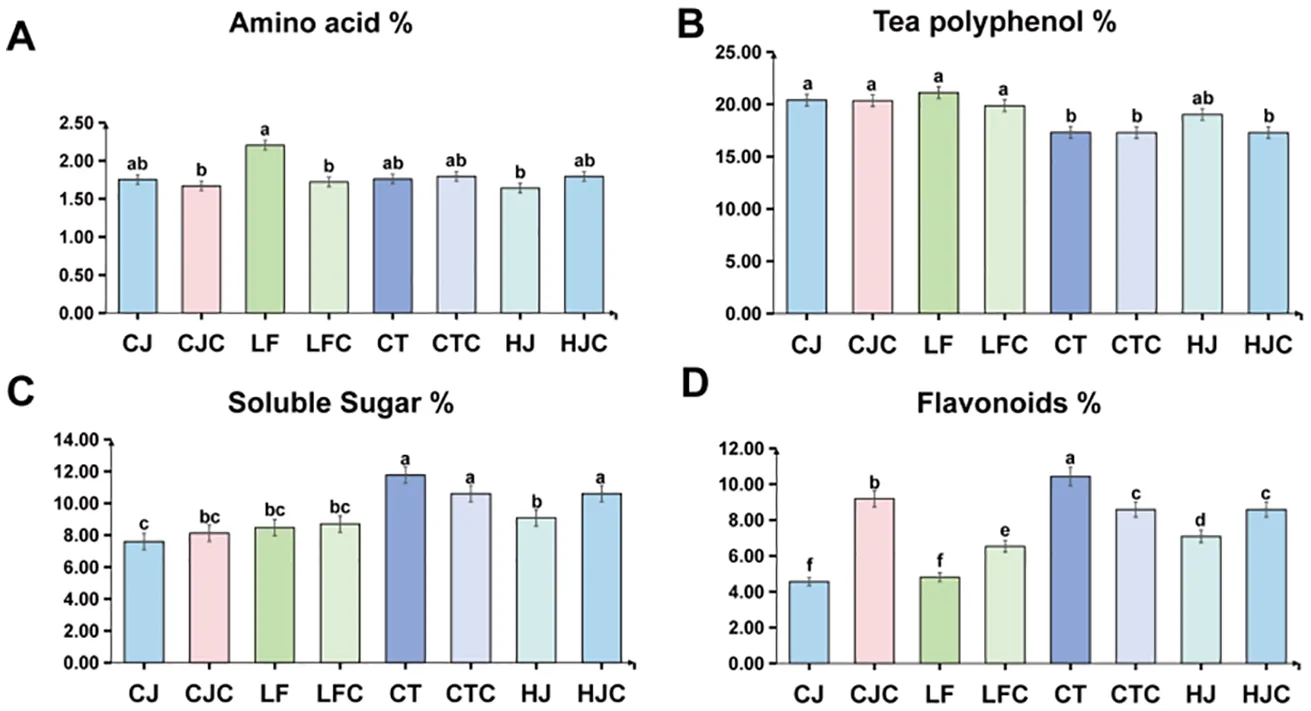
Effects of different intercropping systems on the biochemical quality traits of tea leaves. **(A)** Amino acid content; **(B)** tea polyphenol content; **(C)** soluble sugar content; and **(D)** flavonoid content. Data are presented as mean ± SD (n = 3). Statistical differences were evaluated using two-way ANOVA followed by Tukey’s HSD test. Different lowercase letters indicate significant differences at P < 0.05; bars sharing at least one letter are not significantly different. CT, tea intercropped with *Paris polyphylla*; CTC, the corresponding independent monoculture tea control; LF, tea intercropped with leguminous green manure; LFC, the corresponding independent monoculture tea control; CJ, tea intercropped with *Pleurotus ostreatus*; CJC, the corresponding independent monoculture tea control; HJ, tea intercropped with *Polygonatum kingianum*; and HJC, the corresponding independent monoculture tea control.

Two-way ANOVA revealed trait-specific responses of tea-leaf biochemical properties to intercropping configuration and cropping system (**Figure 1**; **Supplementary Tables S1** and **S2**). A significant interaction between the two factors was detected for free amino acid content (P = 0.031), with LF exhibiting a significantly higher mean than LFC (2.20% vs. 1.72%; Tukey-adjusted P = 0.049). Tea polyphenol content was significantly affected by intercropping configuration (P < 0.001) and cropping system (P = 0.036), whereas their interaction was not significant (P = 0.169). Among the four paired comparisons, HJ showed the largest numerical increase relative to HJC (19.02% vs. 17.30%), corresponding to an increase of approximately 10.0%; however, this difference was not statistically significant (Tukey-adjusted P = 0.197). Soluble sugar content was significantly affected by intercropping configuration and the configuration × cropping system interaction (P < 0.001 and P = 0.005, respectively), whereas the main effect of cropping system was not significant (P = 0.173). CT had the highest mean soluble sugar content but did not differ significantly from CTC (11.77% vs. 10.71%; Tukey-adjusted P = 0.267). In contrast, HJ had a significantly lower soluble sugar content than HJC (9.08% vs. 10.61%; Tukey-adjusted P = 0.039). Flavonoid content was significantly affected by intercropping configuration, cropping system, and their interaction (all P < 0.001). Compared with their respective monoculture controls, CT had a higher flavonoid content (10.43% vs. 8.62%), whereas LF (4.81% vs. 6.53%), CJ (4.57% vs. 9.18%), and HJ (7.08% vs. 8.59%) had lower flavonoid contents (all Tukey-adjusted P < 0.001).

### 3.2 Variations in soil physicochemical properties across intercropping systems

Two-way ANOVA revealed trait-specific effects of intercropping configuration and cropping system on soil physicochemical properties (**Table 1**; **Supplementary Table S3**). Soil pH was significantly affected by intercropping configuration, cropping system, and their interaction (P < 0.001, P = 0.004, and P < 0.001, respectively). HJ had a significantly higher soil pH than HJC (5.69 vs. 4.60; Tukey-adjusted P < 0.001), whereas the other three intercropping treatments did not differ significantly from their corresponding monoculture controls. Significant interaction effects were also detected for AP and TP (both P < 0.001). AP was significantly higher in LF than in LFC (28.84 vs. 2.26 mg kg^−1^) and in CJ than in CJC (12.47 vs. 5.11 mg kg^−1^; both Tukey-adjusted P < 0.001), whereas CT and HJ did not differ significantly from their respective controls. Similarly, TP was significantly higher in LF than in LFC (0.80 vs. 0.46 g kg^−1^, Tukey-adjusted P < 0.001), with no significant differences in the other matched comparisons. Intercropping configuration had significant main effects on SOM, TN, TK, and CEC (all P < 0.001). Among the eight treatment combinations, CJ and CJC had the highest mean SOM and CEC values and the lowest mean TK contents. Cropping system had significant main effects on AK and CEC (both P = 0.005), and CJ had a significantly higher AK content than CJC (573.29 vs. 152.82 mg kg^−1^; Tukey-adjusted P = 0.043). Despite the significant overall cropping-system effect on CEC, none of the individual intercropping–control comparisons for SOM, TN, TK, or CEC reached statistical significance after Tukey adjustment (P > 0.05).

**Table 1:** Physicochemical profiles of the soil under diverse intercropping regimens.

Sample	pH	SOM (g/kg)	TN (%)	AP (mg/kg)	AK (mg/kg)	TK (g/kg)	TP (g/kg)	CEC (cmol/kg(+))
CT	4.73 ± 0.19c	45.64 ± 3.14b	0.25 ± 0.02abc	77.13 ± 29.68a	188.32 ± 83.95ab	20.74 ± 1.19a	0.69 ± 0.06a	13.73 ± 0.76b
CTC	4.57 ± 0.29c	40.07 ± 11.57b	0.23 ± 0.04cd	93.20 ± 25.09a	182.50 ± 75.13ab	21.13 ± 0.41a	0.64 ± 0.06a	12.24 ± 1.46b
LF	4.95 ± 0.12bc	37.77 ± 4.83b	0.20 ± 0.04cd	28.84 ± 3.73a	244.23 ± 128.04ab	17.26 ± 1.10b	0.80 ± 0.08a	12.53 ± 1.28b
LFC	5.37 ± 0.12ab	33.33 ± 4.42b	0.16 ± 0.01d	2.26 ± 0.30d	191.67 ± 34.98ab	17.86 ± 0.94b	0.46 ± 0.03b	10.84 ± 0.78b
CJ	4.76 ± 0.15bc	91.05 ± 9.17a	0.33 ± 0.04ab	12.47 ± 1.89b	573.29 ± 347.35a	6.23 ± 0.53c	0.78 ± 0.03a	21.21 ± 0.97a
CJC	4.37 ± 0.29c	86.61 ± 6.54a	0.33 ± 0.04a	5.11 ± 0.56c	152.82 ± 17.52b	5.99 ± 0.10c	0.62 ± 0.04a	18.95 ± 0.86a
HJ	5.69 ± 0.15a	50.74 ± 16.84b	0.24 ± 0.05abcd	95.55 ± 71.71a	395.17 ± 43.61ab	19.08 ± 1.52ab	0.81 ± 0.26a	14.31 ± 3.06b
HJC	4.60 ± 0.35c	43.48 ± 6.18b	0.23 ± 0.02bcd	95.89 ± 15.16a	189.80 ± 91.85ab	21.38 ± 0.53a	0.66 ± 0.02a	12.17 ± 0.87b

Values are presented as the mean ± standard deviation (n = 3). Means within the same column that do not share a common lowercase letter are significantly different according to Tukey’s HSD test following two-way ANOVA (P < 0.05). CT, tea intercropped with *Paris polyphylla*; CTC, the corresponding independent monoculture tea control; LF, tea intercropped with leguminous green manure; LFC, the corresponding independent monoculture tea control; CJ, tea intercropped with *Pleurotus ostreatus*; CJC, the corresponding independent monoculture tea control; HJ, tea intercropped with *Polygonatum kingianum*; and HJC, the corresponding independent monoculture tea control.

### 3.3 Shifts in rhizosphere microbial α-diversity across distinct intercropping systems

**Figure 2:**
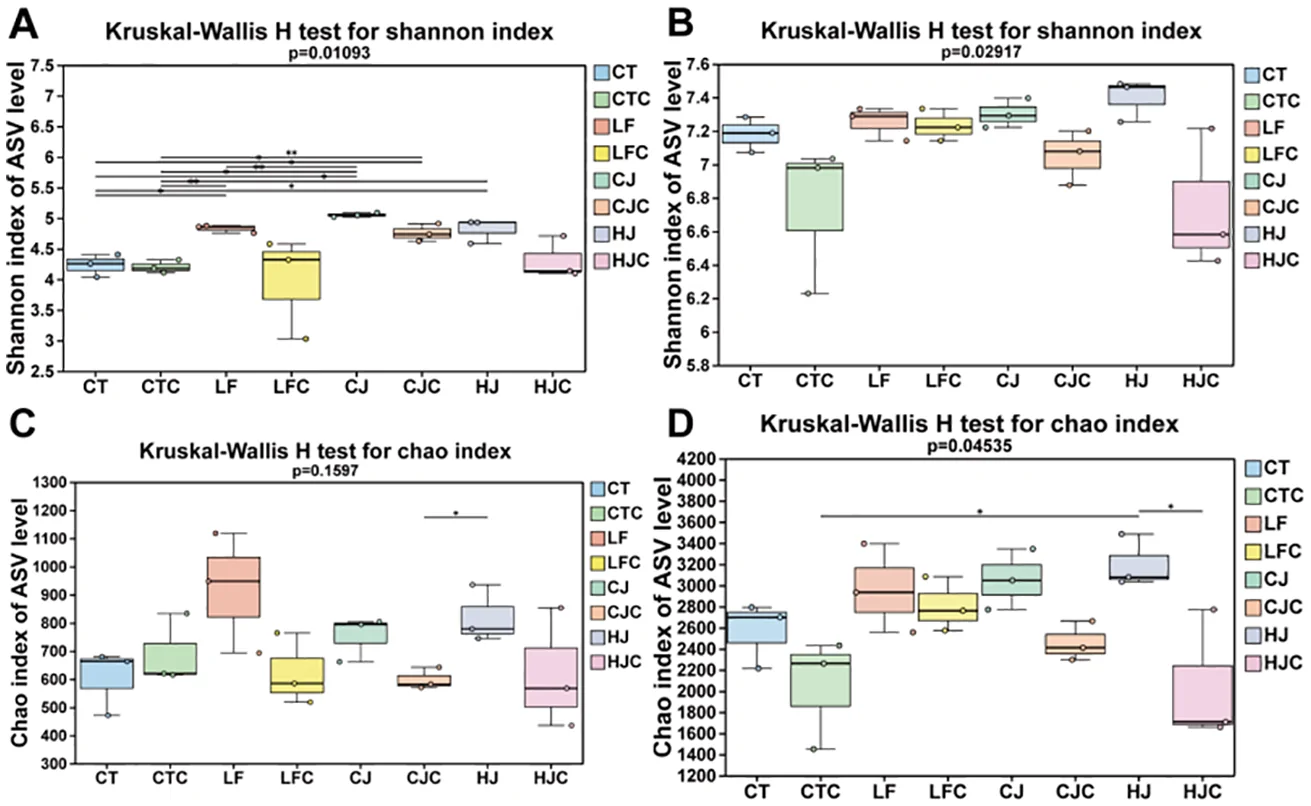
Effects of different intercropping systems on rhizosphere microbial α-diversity. **(A)** Fungal Shannon index; **(B)** bacterial Shannon index; **(C)** fungal Chao index; and **(D)** bacterial Chao index. Boxplots show the median, interquartile range, and individual biological replicates (n = 3). Overall differences among treatments were evaluated using the Kruskal–Wallis H test, followed, where appropriate, by Dunn’s pairwise comparisons with Bonferroni correction. The overall P-value is shown above each panel. Asterisks indicate significant pairwise differences based on adjusted P-values: *Padj < 0.05; **Padj < 0.01. CT, tea intercropped with *Paris polyphylla*; CTC, the corresponding independent monoculture tea control; LF, tea intercropped with leguminous green manure; LFC, the corresponding independent monoculture tea control; CJ, tea intercropped with *Pleurotus ostreatus*; CJC, the corresponding independent monoculture tea control; HJ, tea intercropped with *Polygonatum kingianum*; and HJC, the corresponding independent monoculture tea control.

Rhizosphere microbial α-diversity responded differently to intercropping configuration and microbial kingdom (**Figure 2**; **Supplementary Table S4**). Significant overall treatment effects were detected for fungal Shannon diversity (Kruskal–Wallis, P = 0.0109), bacterial Shannon diversity (P = 0.0292), and bacterial Chao richness (P = 0.0454), whereas fungal Chao richness did not differ significantly among the eight treatments (P = 0.1597). CJ exhibited the highest mean fungal Shannon index (5.05), while HJ showed the highest mean bacterial Shannon index (7.40) and bacterial Chao richness (3199.71). Dunn–Bonferroni comparisons identified significant pairwise differences in fungal Shannon diversity, as indicated in **Figure 2A**. Although the overall treatment effect on bacterial Shannon diversity was significant, none of the adjusted pairwise comparisons reached statistical significance. For bacterial richness, the Chao index was significantly higher in HJ than in HJC (Dunn–Bonferroni-adjusted P < 0.05). LF had the highest mean fungal Chao richness (919.75), but the overall difference among treatments was not significant. Collectively, these results demonstrate that microbial α-diversity responses were configuration- and kingdom-specific rather than uniformly enhanced by intercropping.

### 3.4 Reshaping of rhizosphere microbial assemblages under diverse intercropping regimens

**Figure 3:**
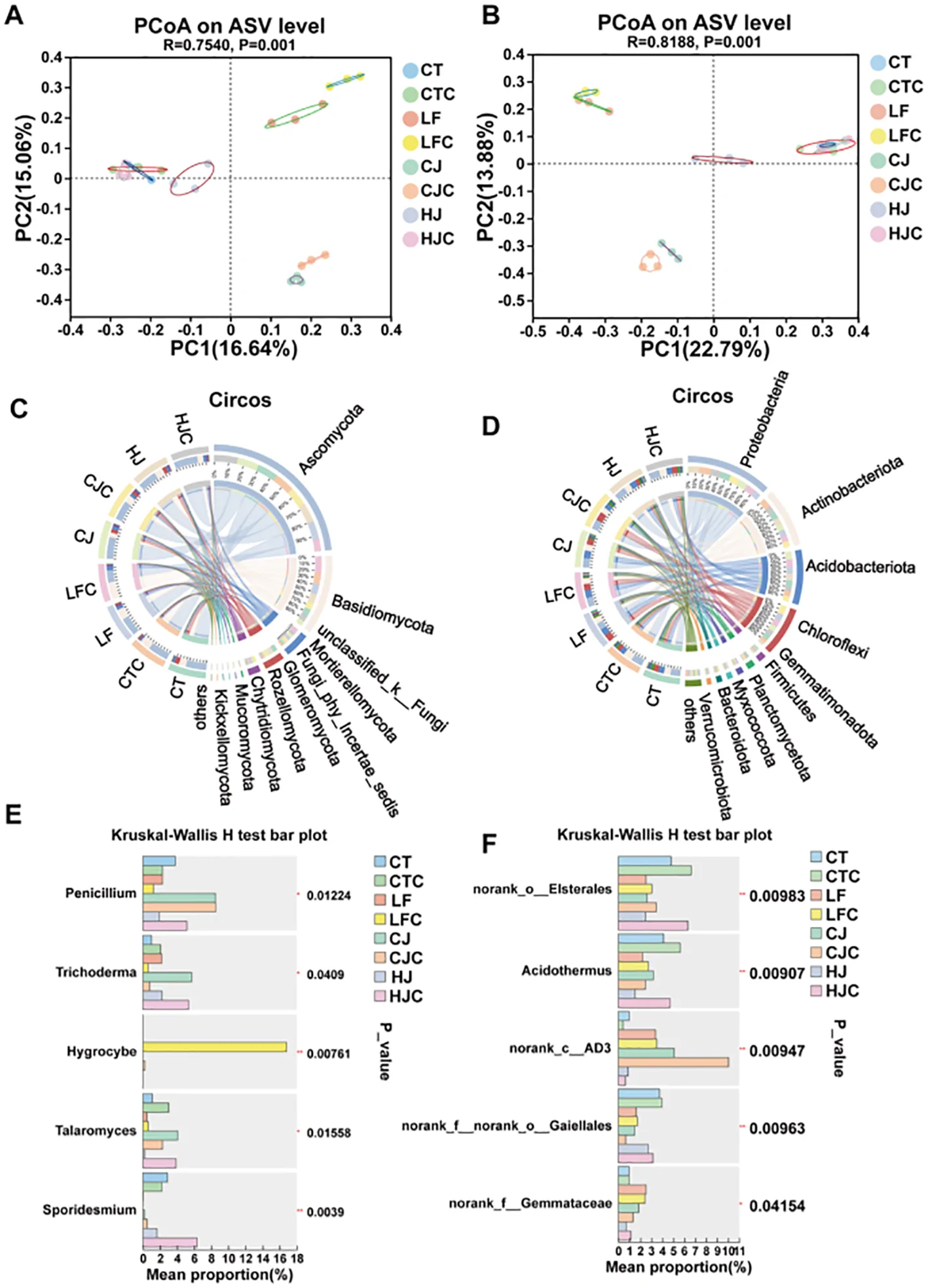
Structural assembly patterns of rhizosphere microbiota in response to distinct intercropping systems. **(A)** PCoA of fungal communities. **(B)** PCoA of bacterial communities. **(C)** Circos plot of fungal community composition at the phylum level. **(D)** Circos plot of bacterial community composition at the phylum level. **(E)** Bar plot showing differences in dominant fungal genera. **(F)** Bar plot showing differences in dominant bacterial genera. CT, tea intercropped with *Paris polyphylla*; CTC, the corresponding independent monoculture tea control; LF, tea intercropped with leguminous green manure; LFC, the corresponding independent monoculture tea control; CJ, tea intercropped with *Pleurotus ostreatus*; CJC, the corresponding independent monoculture tea control; HJ, tea intercropped with *Polygonatum kingianum*; and HJC, the corresponding independent monoculture tea control.

As depicted by the PCoA ordination, the distinct intercropping regimens significantly reshaped the community architecture of the rhizosphere microbiota (**Figure 3**). For the fungal community (**Figure 3A**), the first two axes of the PCoA plot captured 16.64% (PC1) and 15.06% (PC2) of the overall community variance. The CT treatment samples were mainly concentrated on the left side, the LF treatment on the upper right, the CJ treatment on the lower left, and clearly separated from other groups, while the HJ treatment was concentrated in the center-left area (PERMANOVA, R² = 0.7540, *P* = 0.001). For the bacterial community (**Figure 3B**), the primary and secondary axes of the ordination plot captured 22.79% (PC1) and 13.88% (PC2) of the overall community variance. CT treatment samples were distributed in the upper right, LF treatment in the upper left, CJ treatment in the lower left, and HJ treatment in the middle, with significant separation among groups (R² = 0.8188, *P* = 0.001).

Phylum-level community composition analysis (**Figure 3C**) showed that the dominant fungal phyla were Ascomycota (33.71%–67.46%), Basidiomycota (13.14%–48.11%), and Mortierellomycota (0.97%–12.47%), while the dominant bacterial phyla were Proteobacteria (20.05%–33.82%), Acidobacteriota (16.45%–27.04%), Actinobacteriota (14.65%–22.10%), Chloroflexi (9.00%–25.59%), and Gemmatimonadota (1.41%–3.69%). The implementation of intercropping reshaped the abundance profiles of the prevalent bacterial and fungal phyla within the rhizosphere. For fungi, Basidiomycota abundance in CT was significantly lower than in CTC (20.53% vs 30.76%, *P* < 0.05), while Mortierellomycota was significantly higher (12.47% vs 3.72%, *P* < 0.05). In LF, Ascomycota abundance was significantly higher than in LFC (52.29% vs 33.71%, *P* < 0.05), whereas Basidiomycota was significantly lower (29.93% vs 48.11%, *P* < 0.05).

In CJ, Ascomycota abundance was significantly higher than in CJC (61.39% vs 54.31%, *P* < 0.05). In HJ, Basidiomycota abundance was significantly higher than in HJC (25.02% vs 16.85%, *P* < 0.05), while Ascomycota abundance was significantly lower (53.46% vs 67.46%, *P* < 0.05). For bacteria (**Figure 3D**), Proteobacteria and Actinobacteriota abundances in CT were significantly lower than in CTC (28.47% vs 33.28%, 19.40% vs 27.04%, *P* < 0.05), whereas Acidobacteriota and Chloroflexi were significantly higher (16.65% vs 14.88%, 14.46% vs 10.02%, *P* < 0.05). In LF, Proteobacteria abundance was significantly lower than in LFC (20.05% vs 26.63%, *P* < 0.05), and Chloroflexi abundance was significantly higher (21.68% vs 18.41%, *P* < 0.05). In CJ, Acidobacteriota abundance was significantly higher than in CJC (22.10% vs 14.65%, *P* < 0.05), while Chloroflexi was significantly lower (16.32% vs 25.59%, *P* < 0.05). In HJ, Proteobacteria and Acidobacteriota abundances were significantly higher than in HJC (33.82% vs 31.19%, 19.04% vs 15.71%, *P* < 0.05), whereas Actinobacteriota and Chloroflexi were significantly lower (19.10% vs 21.12%, 9.00% vs 15.42%, *P* < 0.05).

At the genus level, different intercropping systems significantly altered the composition of rhizosphere fungal and bacterial communities in tea plants. The dominant fungal genera (**Figure 3E**) included *Penicillium*, *Trichoderma*, *Talaromyces*, *Sporidesmium*, and *Knufia*. In CT intercropping, the fungal genera *Penicillium* and *Sporidesmium* were markedly enriched relative to the CTC baseline (*P* < 0.05). LF intercropping markedly enriched *Penicillium*, *Trichoderma*, *Sporidesmium*, and *Knufia* (*P* < 0.05). In CJ intercropping, the abundances of *Trichoderma*, *Talaromyces*, and *Knufia* were markedly higher than in CJC (*P* < 0.05). In contrast, in HJ intercropping, the abundances of these dominant fungal genera were all markedly lower than in HJC (*P* < 0.05).

The dominant bacterial genera (**Figure 3F**) mainly included norank_o:Elsterales, *Acidothermus*, norank_c:AD3, norank_o:Gaiellales, and norank_f:Gemmataceae. In CT intercropping, norank_c:AD3 and norank_f:Gemmataceae were significantly more abundant than in CTC (*P* < 0.05). In LF intercropping, norank_f:Gemmataceae significantly exceeded the levels recorded in the LFC control (*P* < 0.05). In CJ intercropping, *Acidothermus*, norank_o:Gaiellales, and norank_f:Gemmataceae were significantly more abundant than in CJC (*P* < 0.05). The relative frequency of norank_c:AD3 was markedly enriched in the HJ intercropping system compared to the HJC control (*P* < 0.05).

### 3.5 Differential microbial taxa screening under different intercropping systems

**Figure 4:**
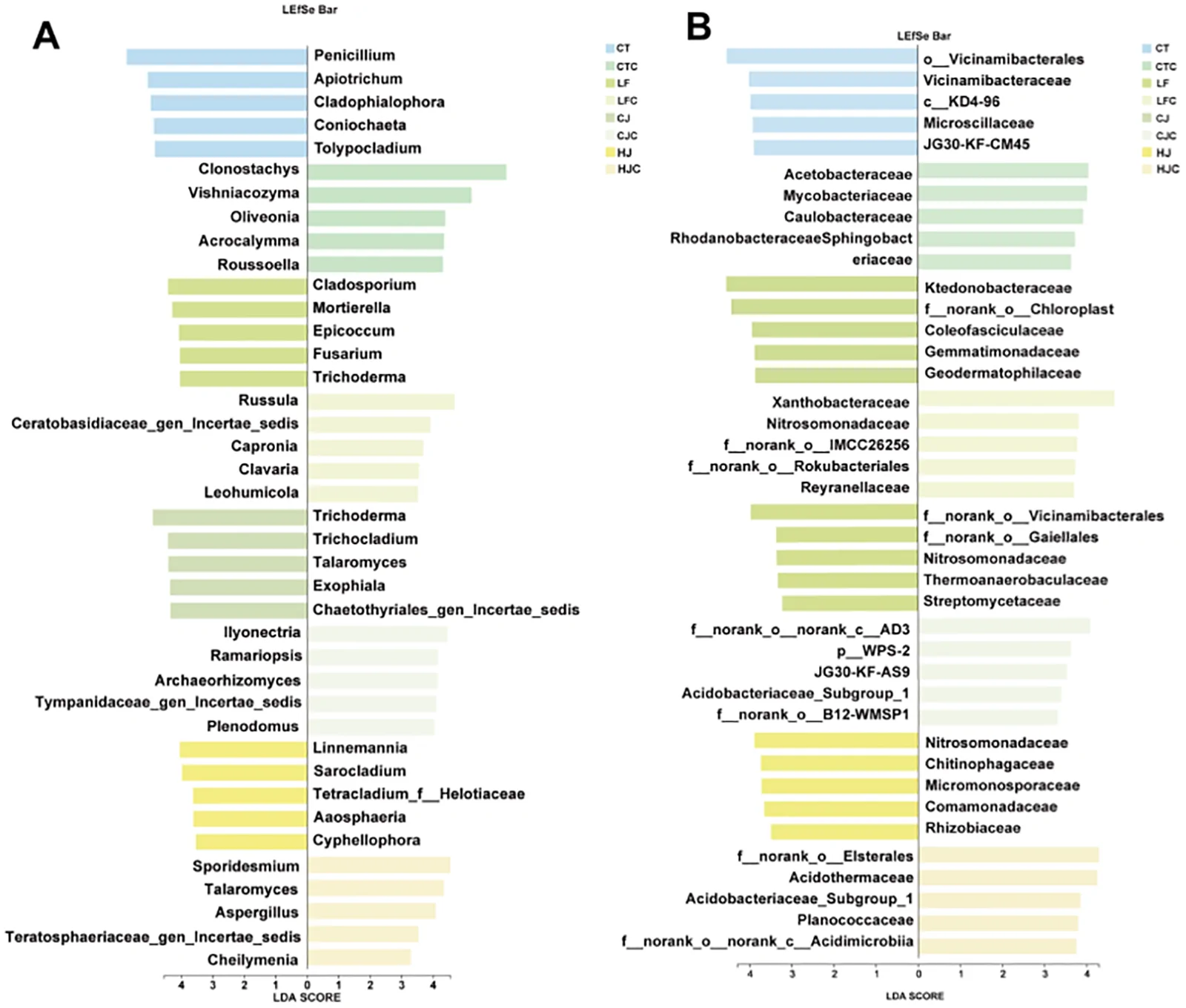
Differential microbial biomarkers were identified under different intercropping systems using LEfSe analysis. **(A)** Fungal biomarkers analyzed at the genus level; **(B)** bacterial biomarkers analyzed at the family level. For bacterial features that could not be confidently assigned to a formally named family, the deepest reliably classified parental taxon is shown. Bar length represents the linear discriminant analysis (LDA) score, and bar color indicates the treatment in which the taxon was enriched. Only taxa with an LDA score ≥ 3.0 and P < 0.05 are displayed. CT, tea intercropped with Paris polyphylla; CTC, the corresponding independent monoculture tea control; LF, tea intercropped with leguminous green manure; LFC, the corresponding independent monoculture tea control; CJ, tea intercropped with *Pleurotus ostreatus*; CJC, the corresponding independent monoculture tea control; HJ, tea intercropped with *Polygonatum kingianum*; and HJC, the corresponding independent monoculture tea control.

The LEfSe algorithm identified distinct microbial biomarkers that were significantly enriched across the various intercropping regimens (LDA ≥ 3, *P* < 0.05; **Figure 4A**). For fungal differential genera, CT treatment was enriched with *Penicillium*, *Apiotrichum*, and *Cladophialophora*. LF treatment was enriched with *Cladosporium*, *Mortierella*, *Epicoccum*, *Fusarium*, and *Trichoderma*. CJ treatment was enriched with *Trichoderma*, *Trichocladium*, *Talaromyces*, and *Exophiala*. HJ treatment was enriched with *Linnemannia*, *Sarocladium*, and *Tetracladium.* For bacterial differential families (**Figure 4B**), CT treatment was enriched with Vicinamibacteraceae, Microscillaceae, and JG30-KF-CM45. LF treatment was enriched with Ktedonobacteraceae, Coleofasciculaceae, Gemmatimonadaceae, and Geodermatophilaceae. The CJ treatment was enriched with Nitrosomonadaceae, Thermoanaerobaculaceae, and Streptomycetaceae. HJ treatment was enriched with Nitrosomonadaceae, Chitinophagaceae, Micromonosporaceae, Comamonadaceae, and Rhizobiaceae.

### 3.6 Associations between soil physicochemical properties, microbial communities, and tea quality

The structural assembly of soil microbiota was significantly influenced by physicochemical attributes. For the bacterial sector, factors including AP (r = 0.592), TK (r = 0.400), pH (r = 0.380), TN (r = 0.191), and TP (r = 0.190) all demonstrated significantly positive correlations (*P* < 0.05). However, the observed fluctuations in SOM, AK, and CEC lacked sufficient statistical evidence to be considered significant (*P* > 0.05). Drawing a parallel, the fungal community composition was positively driven by TN, TK, TP, AP, and pH (with r values ranging from 0.214 to 0.400, *P* < 0.05), while no significant linkages were identified with SOM, AK, and CEC (r = −0.058–0.180, *P* > 0.05).

**Figure 5:**
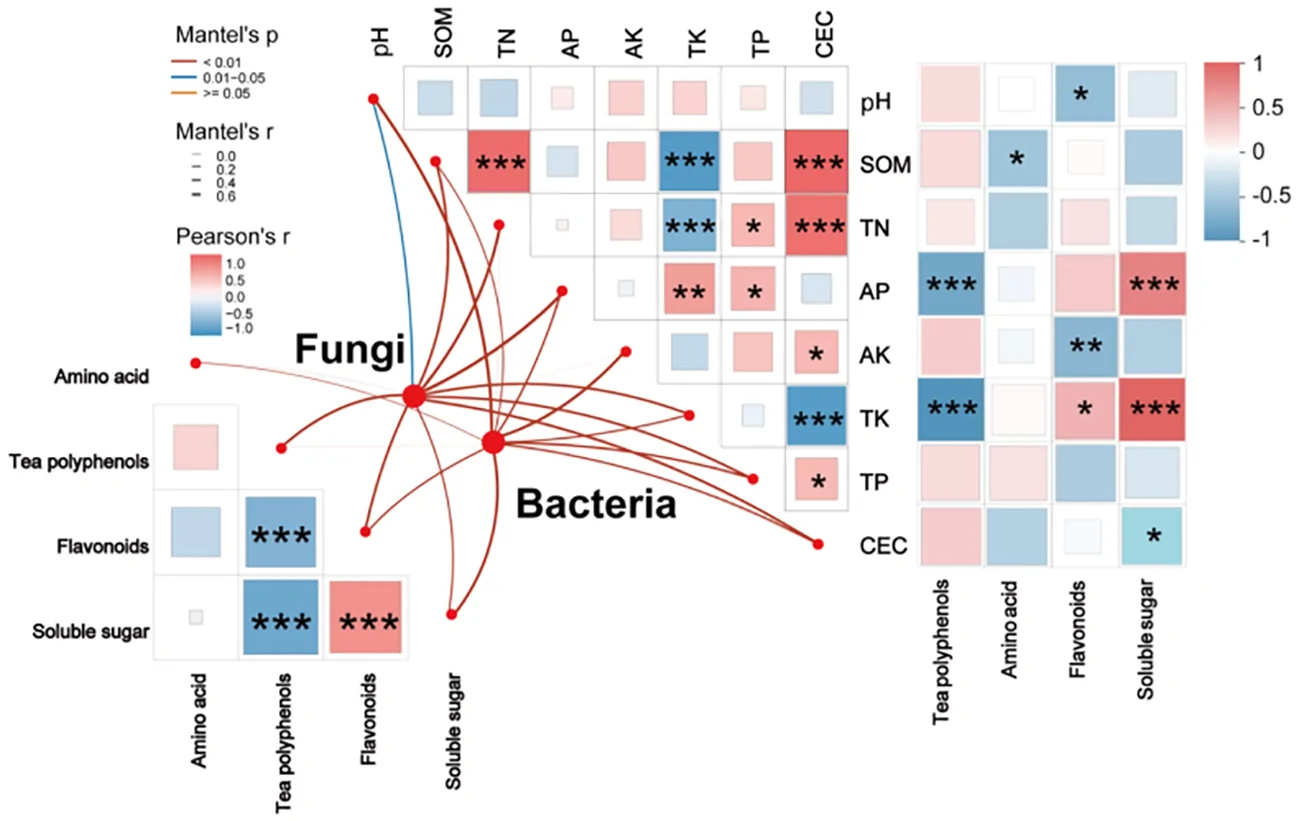
Correlations among rhizosphere microbial communities, tea quality, and soil properties. **(A)** Mantel-test heatmap: lines indicate correlations between communities and environmental factors (line thickness ∝ |Mantel’s r|, with positive or negative relationships shown. Heatmap colors reflect correlation direction and strength; asterisks denote significance: (*, **, ***) denote statistical significance levels at P ≤ 0.05, P ≤ 0.01, and P ≤ 0.001, respectively. CT, tea intercropped with *Paris polyphylla*; CTC, the corresponding independent monoculture tea control; LF, tea intercropped with leguminous green manure; LFC, the corresponding independent monoculture tea control; CJ, tea intercropped with *Pleurotus ostreatus*; CJC, the corresponding independent monoculture tea control; HJ, tea intercropped with *Polygonatum kingianum*; and HJC, the corresponding independent monoculture tea control.

Pearson correlation analysis identified several significant associations between soil properties and tea biochemical traits (**Figure 5**). Tea polyphenol content was negatively correlated with AP (r = −0.610, P = 0.0016) and TK (r = −0.629, P = 0.0010). Soluble sugar content was negatively correlated with SOM (r = −0.524, P = 0.0086) and CEC (r = −0.529, P = 0.0079), but positively correlated with AP (r = 0.615, P = 0.0014) and TK (r = 0.744, P < 0.001). Flavonoid content was negatively correlated with AK (r = −0.492, P = 0.0147). No significant correlations were detected between free amino acid content and the measured soil physicochemical properties (P > 0.05).

### 3.7 Predicted functional profiling of soil microorganisms via the PICRUSt2 pipeline

**Figure 6:**
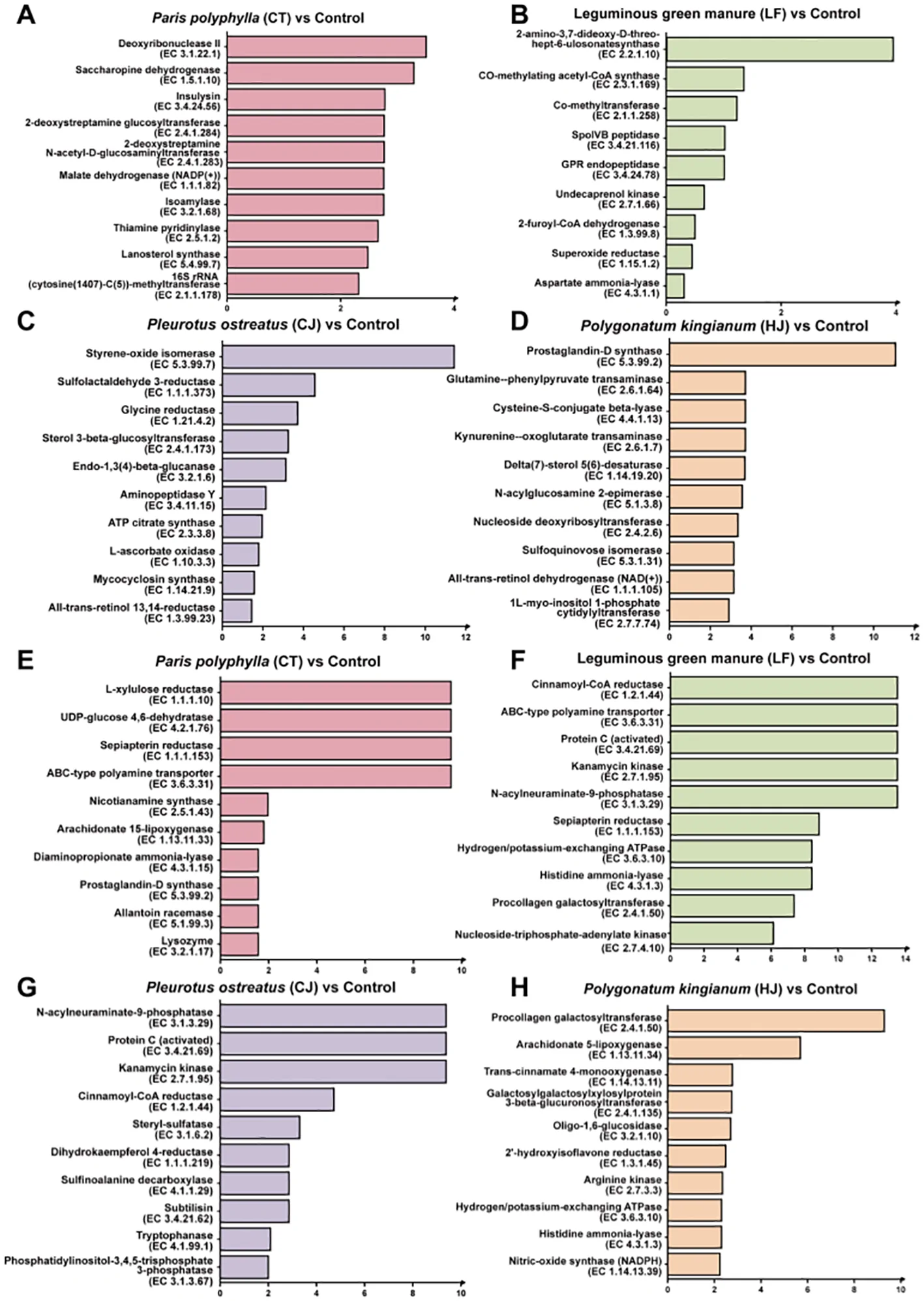
The top 10 significantly upregulated functional enzymes in rhizosphere microbial communities under different intercropping modes predicted by PICRUSt2. Bar length represents the fold increase in predicted enzyme abundance in each intercropping treatment relative to its corresponding monoculture control (Log_2_FC). The y-axis shows the specific Enzyme Commission (EC) numbers. **(A–D)** Bacterial community based on 16S rRNA sequencing. **(E–H)** Fungal community based on ITS sequencing. Intercropping modes include CT, LF, CJ, and HJ. CT, tea intercropped with *Paris polyphylla*; CTC, the corresponding independent monoculture tea control; LF, tea intercropped with leguminous green manure; LFC, the corresponding independent monoculture tea control; CJ, tea intercropped with *Pleurotus ostreatus*; CJC, the corresponding independent monoculture tea control; HJ, tea intercropped with *Polygonatum kingianum*; and HJC, the corresponding independent monoculture tea control.

Functional attributes of the soil microbiomes were predicted based on the taxonomic information retrieved from 16S and ITS sequencing datasets; PICRUSt2 was applied to predict enzyme functions (Enzyme Commission, EC) of rhizosphere bacterial and fungal communities under different intercropping modes, and differential analyses were conducted (**Figure 6**) to characterize intercropping-driven changes in the potential ecological functions of microbial communities. It is essential to recognize the distinction between PICRUSt2-based functional inference and the direct measurement of gene expression or enzymatic kinetics, as the former reflects genomic potential derived from marker gene profiles rather than actual metabolic activity; therefore, these results should be interpreted cautiously and used only as supporting evidence for mechanistic inference. In addition, ITS-based functional inference is more strongly influenced by factors such as reference genome coverage, and thus interpretation should avoid overly causal statements.

#### 3.7.1 Functional prediction results of the bacterial community (16S)

Compared with the monoculture controls, all four intercropping modes significantly altered the functional potential of rhizosphere bacteria (**Figures 6A–D**) and exhibited mode-specific enrichment patterns of metabolic enzymes (*P <* 0.05).

CT mode: CT showed significant enrichment of enzymes related to carbon metabolism and energy turnover (**Figure 6A**), such as NADP^+^-dependent oxidative decarboxylating malic enzyme (EC 1.1.1.82, Log_2_FC = 2.76) and isoamylase (EC 3.2.1.68). In addition, saccharopine dehydrogenase (NADP^+^, L-glutamate-forming; EC 1.5.1.10), which is involved in lysine/amino acid metabolism, was also significantly enriched, suggesting that the CT rhizosphere bacterial community may have more active potential for carbon flux conversion and transformation of nitrogen-containing organic matter.

LF mode: LF significantly enriched enzymes associated with coupled carbon–nitrogen metabolism and energy transformation, such as CO-methylating acetyl-CoA synthase (EC 2.3.1.169, Log_2_FC = 1.35), which is related to acetyl-CoA generation/one-carbon metabolism, and aspartate ammonia-lyase (EC 4.3.1.1), which participates in amino acid degradation and nitrogen turnover. These results suggest that the rhizosphere microbiome in LF may be more inclined to promote carbon and nitrogen transformation and turnover processes.

CJ mode: CJ significantly upregulated key enzymes related to organic carbon conversion and metabolic energy supply, such as ATP-citrate lyase (EC 2.3.3.8, Log_2_FC = 1.96) and glycine reductase (EC 1.21.4.2). This pattern is consistent with increased exogenous organic substrate inputs following the incorporation of mushroom residues, suggesting that the CJ rhizosphere bacterial community may have stronger potential for carbon source utilization and reductive metabolism, thereby accelerating organic substrate turnover and influencing nutrient availability.

HJ mode: HJ significantly enriched multiple core enzymes involved in amino acid metabolism and carbohydrate conversion, including glutamine aminotransferase (EC 2.6.1.64, Log_2_FC = 3.72), kynurenine aminotransferase (EC 2.6.1.7), and sulfoquinovose isomerase (EC 5.3.1.31). These results indicate that the HJ rhizosphere bacterial community has a relatively high potential for nitrogen-containing organic matter conversion and carbohydrate metabolism, which may be associated with nutrient acquisition and metabolic allocation processes in tea plants.

#### 3.7.2 Functional prediction results of the fungal community (ITS)

ITS-based functional prediction (**Figures 6E–H**) indicated that intercropping modes could also generate differentiated functional tendencies at the fungal level, with significant differences (*P <* 0.05) particularly in carbohydrate metabolism, amino acid transformation, and certain secondary-metabolism-related reactions. Given the uncertainty of ITS-based inference, these results are mainly used to depict potential functional directions and to corroborate community-level differences.

CT mode: In CT, enzymes related to carbohydrate metabolism were markedly upregulated in the fungal community, such as L-xylulose reductase (EC 1.1.1.10) and UDP-glucose 4,6-dehydratase (EC 4.2.1.76) (Log_2_FC > 9.0). This suggests that CT rhizosphere fungi may have a stronger potential for complex carbohydrate conversion, thereby providing more available carbon sources and metabolic intermediates in the rhizosphere.

LF mode: LF showed significant enrichment of histidine ammonia-lyase (EC 4.3.1.3, Log_2_FC = 8.42) and nucleoside-triphosphate adenylyltransferase (EC 2.7.4.10), indicating enhanced potential for amino acid transformation and energy metabolism in the fungal community, which may be related to the observed phenotype of promoted nitrogen assimilation and amino acid accumulation.

CJ mode: CJ significantly upregulated multiple enzyme signals associated with substrate transformation and secondary metabolism, such as dihydrokaempferol 4-reductase (EC 1.1.1.219, Log_2_FC = 2.86) and tryptophanase (EC 4.1.99.1). These results suggest that the CJ rhizosphere fungal community may have higher metabolic diversity and substrate transformation capacity; however, its short-term effects on tea quality still require integrated evaluation together with soil physicochemical indicators and metabolomic evidence.

HJ mode: In HJ, trans-cinnamate 4-monooxygenase (EC 1.14.13.11, Log_2_FC = 2.77) was significantly upregulated. This enzyme is one of the key reaction enzymes in the phenylpropanoid pathway, catalyzing the conversion of cinnamate to p-coumarate, suggesting enhanced functional potential of HJ rhizosphere fungi in phenylpropanoid-related reactions. In addition, histidine ammonia-lyase (EC 4.3.1.3) and arginine kinase (EC 2.7.3.3) were also significantly enriched, indicating that the HJ fungal community may simultaneously possess strong potential for amino acid metabolism and energy transformation.

## 4 Discussion

### 4.1 Mode-dependent effects of intercropping on soil physicochemical properties

The soil data indicate that intercropping did not uniformly improve soil fertility; instead, the direction and magnitude of the responses depended on the identity of the intercropping partner. Among the four systems, HJ produced the clearest change in soil acidity, with soil pH being significantly higher than in HJC. Soil pH is an important regulator of nutrient solubility, elemental uptake, and tea-leaf quality (Jia et al., 2024). The significant associations of pH with both bacterial and fungal community dissimilarities further suggest that the less acidic conditions in HJ may have acted as an environmental filter for rhizosphere community assembly. This interpretation is consistent with evidence that soil pH is a major determinant of bacterial and fungal community composition (Rousk et al., 2010). Nevertheless, because root-exudate composition, proton fluxes, nitrification rates, and exchangeable Al were not measured, the higher pH in HJ cannot be attributed directly to specific compounds released by *Polygonatum kingianum*. Altered root ion balance, organic-acid turnover, and microbially mediated nutrient transformations represent plausible but currently unverified mechanisms (Hinsinger et al., 2003; Korenblum et al., 2022).

LF showed a distinct phosphorus-related response, with both AP and TP being significantly higher than in LFC. Leguminous green manure can affect soil P pools through rhizodeposition, root turnover, residue decomposition, microbial P immobilization and release, phosphatase activity, and the vertical redistribution of nutrients from deeper soil layers (Duan et al., 2024; Hallama et al., 2019). These processes may have contributed to the higher AP and TP values measured in LF, although their relative contributions could not be distinguished in the present study. In contrast, TN did not differ significantly between LF and LFC, and biological N fixation was not measured. Therefore, the higher free amino acid content of tea leaves in LF should not be attributed directly to an experimentally demonstrated increase in soil N input. Because plant–microbe competition and microbial turnover can alter access to rapidly cycling N pools without necessarily causing a detectable change in bulk-soil TN, the LF response may instead involve short-term N turnover, organic-N availability, root N uptake, or plant C–N allocation (Kuzyakov and Xu, 2013).

CJ significantly increased AP and AK relative to CJC. The cultivation substrate and returned residues associated with *Pleurotus ostreatus* may have supplied P and K while also providing lignocellulosic material for microbial decomposition. Residual fungal biomass and lignocellulolytic enzymes in spent mushroom substrate may further facilitate organic-matter decomposition and nutrient release (Phan and Sabaratnam, 2012). Previous research on tea–*P. ostreatus* intercropping has similarly demonstrated changes in soil nutrient conditions and rhizosphere microbial community structure (Yang et al., 2024). In the present study, however, CJ did not significantly increase SOM or CEC relative to CJC. Therefore, CJ should be interpreted as primarily modifying labile P and K pools rather than producing a general increase in soil organic matter or nutrient-retention capacity. The relatively large variation in AK under CJ also indicates that this response should be confirmed using additional plots and sampling periods.

In contrast, CT did not differ significantly from CTC in any of the measured soil properties. Therefore, the higher flavonoid content observed in CT cannot be attributed to corresponding changes in the measured bulk-soil nutrient variables. Unmeasured rhizosphere processes, including root exudation, microscale nutrient gradients, and microbially transformed metabolites, may generate chemical conditions that are not adequately represented by bulk-soil measurements (Korenblum et al., 2022). Microclimatic variation and altered plant carbon allocation may also have contributed, but these factors were not quantified and therefore remain hypotheses requiring further investigation. Collectively, the four intercropping systems produced distinct edaphic trajectories: reduced soil acidity in HJ, higher P pools in LF, increased labile P and K in CJ, and limited changes in the measured bulk-soil properties in CT.

### 4.2 Intercropping-induced restructuring of rhizosphere microbial communities

Microbial responses depended on both the intercropping configuration and the microbial kingdom. Accordingly, the results did not support the general conclusion that all four intercropping systems uniformly increased microbial diversity. CJ exhibited the highest mean fungal Shannon index, whereas HJ contained the greatest number of unique bacterial ASVs. These metrics represent different aspects of community structure and should not be treated as equivalent indicators of ecosystem stability. In particular, microbial stability refers to properties such as resistance and resilience following disturbance, which cannot be inferred solely from Shannon diversity or the number of unique ASVs (Shade et al., 2012). The contrasting responses are consistent with the concept that different plant species and cultivation substrates create distinct rhizosphere resource environments through root-derived compounds, organic residues, and microbial transformation of these materials (Korenblum et al., 2022; Zhalnina et al., 2018).

LEfSe analysis further demonstrated configuration-specific taxonomic selection (Segata et al., 2011). The fungal analysis was performed at the genus level, whereas the bacterial analysis was conducted at the family level because a large proportion of the bacterial ASVs could not be confidently classified at the genus level. CT was characterized by the enrichment of *Penicillium*, whereas LF was associated with *Mortierella* and *Trichoderma*. CJ enriched fungal genera including *Talaromyces* and *Exophiala*. In HJ, bacterial families including Nitrosomonadaceae, Chitinophagaceae, Micromonosporaceae, Comamonadaceae, and Rhizobiaceae were enriched. Some members of *Trichoderma*, for example, have documented roles in plant growth promotion, nutrient transformation, and biological control (Tyśkiewicz et al., 2022). However, such traits are frequently species- or strain-specific, and the phylogenetic conservation of microbial traits differs among functions. Taxonomic enrichment alone therefore does not demonstrate that the corresponding ecological functions were expressed in the present soils (Martiny et al., 2015).

Accordingly, the LEfSe results should primarily be interpreted as identifying candidate microbial groups that responded to the different intercropping environments. Verification of their ecological roles would require targeted isolation and inoculation experiments, quantitative analysis of functional genes, or direct functional measurements using metagenomics, metatranscriptomics, or metabolomics (Knight et al., 2018). Nevertheless, the observed community differences provide a basis for developing testable hypotheses linking configuration-specific soil conditions, rhizosphere microbial selection, and tea-leaf biochemical composition; they should not yet be considered evidence of a causal microbial mechanism.

### 4.3 Mode-specific predicted microbial functional potentials

Marker-gene-based functional inference was used to examine whether the observed taxonomic shifts were accompanied by differences in predicted microbial metabolic potential. Because these profiles are inferred from taxonomic marker genes, they represent estimated gene or enzyme repertoires rather than directly measured gene expression, protein abundance, or enzyme activity (Djemiel et al., 2022; Douglas et al., 2020). This limitation is particularly important for ITS-based fungal functional inference, for which assignment accuracy depends strongly on reference-genome coverage and the annotation algorithm used (Krivonos et al., 2023).

In CT, L-xylulose reductase (EC 1.1.1.10), UDP-glucose 4,6-dehydratase (EC 4.2.1.76), and NADP^+^-dependent malate dehydrogenase (EC 1.1.1.82) showed higher predicted relative abundances than in CTC. The annotated functions of these enzymes are broadly associated with carbohydrate conversion and redox reactions (Chang et al., 2021), suggesting a shift in the predicted potential for carbon-substrate transformation rather than demonstrating enhanced metabolic activity. CT had a significantly higher tea-leaf flavonoid content than CTC, whereas the difference in soluble sugar content was numerical. Thus, the predicted functional profile provides a testable hypothesis concerning rhizosphere substrate turnover or plant–microbe signaling but does not establish that microbial carbohydrate metabolism directly promoted sugar or flavonoid biosynthesis in tea leaves. Flavonoid accumulation is ultimately controlled by the plant phenylpropanoid pathway and its associated regulatory and biosynthetic enzymes (Vogt, 2010).

In LF, histidine ammonia-lyase (EC 4.3.1.3), aspartate ammonia-lyase (EC 4.3.1.1), and other amino-acid-related enzymes showed higher predicted relative abundances than in LFC. These enzymes are involved primarily in microbial amino acid degradation or interconversion (Chang et al., 2021) and should not be regarded as direct indicators of enhanced plant nitrogen assimilation. In plants, ammonium assimilation and amino acid production are largely mediated by the glutamine synthetase/glutamate synthase pathway (Masclaux-Daubresse et al., 2010). Nevertheless, the predicted enrichment of microbial amino-acid-transforming functions, together with the LF-associated fungal taxa and the higher free amino acid content of tea leaves, supports the hypothesis that LF altered rhizosphere organic-N turnover or substrate availability to tea roots. These processes may also be influenced by microbial mineralization, immobilization, and competition between roots and microorganisms (Kuzyakov and Xu, 2013). Direct measurements of inorganic and organic N fractions, plant N uptake, and GS/GOGAT activities would be required to verify this pathway.

In HJ, the fungal functional inference assigned a higher predicted relative abundance to trans-cinnamate 4-monooxygenase. This enzyme catalyzes the conversion of cinnamate to *p*-coumarate and is generally characterized as a plant cytochrome P450 involved in phenylpropanoid and flavonoid biosynthesis (Vogt, 2010; Chang et al., 2021). Its identification through an ITS-based fungal prediction should therefore be verified against the exact prediction algorithm, reference sequences, and EC annotation mapping before being interpreted as a fungal function. Even if the assignment is retained, it cannot be equated with phenylpropanoid flux in tea leaves, which is regulated by tea-plant genes and enzyme activities (Liu et al., 2015). Moreover, HJ showed only a numerical increase in tea polyphenol content relative to HJC, without a statistically significant difference in the matched comparison. This predicted profile should therefore be regarded as an annotation-derived hypothesis concerning phenylpropanoid-related substrate transformation rather than evidence that the rhizosphere microbiome stimulated tea polyphenol synthesis.

In CJ, ATP-citrate lyase (EC 2.3.3.8) and other substrate-conversion-related enzymes showed higher predicted relative abundances than in CJC. ATP-citrate lyase links citrate cleavage with the formation of cytosolic acetyl-CoA and oxaloacetate (Chang et al., 2021). Together with the higher mean fungal diversity and the enrichment of *Talaromyces* and *Exophiala*, this profile is consistent with differences in the predicted potential for the transformation of mushroom cultivation substrates. Spent mushroom substrate contains lignocellulosic materials that can support microbial decomposition and nutrient release (Phan and Sabaratnam, 2012). However, the present data cannot distinguish nutrient mineralization from microbial immobilization or plant–microbe competition (Kuzyakov and Xu, 2013). Although CJ significantly increased soil AP and AK, it decreased tea-leaf flavonoid content relative to CJC, indicating that increased soil nutrient availability did not uniformly translate into improved tea biochemical quality.

Overall, these functional predictions identify configuration-associated metabolic hypotheses rather than confirmed mechanisms. CT was associated mainly with predicted carbohydrate-conversion and redox functions, LF with amino acid turnover, HJ with a tentative and annotation-dependent phenylpropanoid-related signal, and CJ with substrate decomposition and acetyl-CoA-related metabolism. These hypotheses should be tested using shotgun metagenomics to verify gene content, metatranscriptomics to evaluate gene expression, metaproteomics and enzyme assays to measure proteins and biochemical activities, and tea-root or leaf metabolomics to establish corresponding plant metabolic responses (Knight et al., 2018; Djemiel et al., 2022).

### 4.4 Integrated soil–microbiome regulation of tea quality

Integration of the soil, microbial, and tea-quality datasets revealed configuration-dependent associations among soil conditions, rhizosphere microbial communities, and tea biochemical traits. Mantel tests showed that bacterial community dissimilarity was significantly associated with variation in tea polyphenols, soluble sugars, and flavonoids, but not with free amino acids. Fungal community dissimilarity was significantly associated with tea polyphenols and flavonoids, but not with free amino acids or soluble sugars. In addition, variation in soil pH, TN, AP, TK, and TP was associated with both bacterial and fungal community composition, whereas SOM, AK, and CEC were not significantly associated with either community. Because these analyses were conducted across all samples, the resulting relationships represent dataset-level covariation rather than effects unique to any individual intercropping configuration. Moreover, Mantel associations do not establish causal direction or microbial mediation.

Pearson correlation analysis revealed trait-specific relationships between soil properties and tea biochemical traits. Tea polyphenol content was negatively correlated with AP and TK, whereas soluble sugar content was positively correlated with AP and TK but negatively correlated with SOM and CEC. Flavonoid content was negatively correlated with AK. No significant correlations were detected between free amino acid content and the measured soil physicochemical properties. These relationships represent dataset-level associations and should not be interpreted as evidence of direct causal effects (Schober et al., 2018).

The contrasting associations of AP and TK with polyphenols and soluble sugars may reflect differences in carbon allocation between primary and secondary metabolic pathways. The negative associations of soluble sugars with SOM and CEC and that of flavonoids with AK may similarly reflect treatment-specific nutrient balance, plant resource allocation, or covariation with other unmeasured environmental factors rather than direct inhibitory effects of these soil properties. The accumulation of amino acids and flavonoids in young tea shoots is closely coordinated with carbon and nitrogen metabolism in roots and mature leaves (Liu et al., 2021). Nevertheless, the present correlations should not be interpreted as evidence that high P or K directly suppresses tea polyphenol synthesis. Under controlled P-supply conditions, P deficiency decreased tea polyphenols, flavonoids, and free amino acids but increased soluble sugars (Lin et al., 2012), demonstrating that the direction of the response depends on nutrient status and experimental conditions. Similarly, a meta-analysis showed that appropriate K fertilization generally increased tea yield and several quality components, including polyphenols and amino acids, although the magnitude of these responses depended on the K application rate and the initial soil available-K status (Xi et al., 2023). The negative TK–polyphenol correlation detected in the present study may therefore reflect treatment-specific covariation, nutrient balance, or other unmeasured environmental factors rather than a universal inhibitory effect of soil K.

The configuration-specific results suggest several testable pathways. In LF, higher AP and TP, enrichment of *Mortierella* and *Trichoderma*, and a predicted functional profile related to amino acid turnover coincided with a significant increase in leaf free amino acids. Leguminous green manure incorporation can modify rhizodeposition, microbial nutrient turnover, and the availability of different soil N pools (Duan et al., 2024). However, free amino acid content was not significantly associated with bacterial or fungal community dissimilarity, and LF did not significantly increase TN relative to LFC. Because soil TN does not directly represent short-term changes in NH_4_^+^, NO_3_^−^, dissolved organic N, or individual amino acids, the LF response may involve direct plant responses to rhizodeposition, short-term organic-N turnover, or unmeasured available-N fractions. These pools are jointly affected by mineralization, microbial immobilization, and root–microbe competition (Kuzyakov and Xu, 2013). Tea roots can also directly absorb soil amino acids through transporters such as CsLHT1 and CsLHT6 (Li et al., 2021), providing a possible pathway that should be tested by measuring soil N fractions, root N uptake, and plant N-assimilation activities.

In CT, the significant increase in flavonoid content occurred without significant changes in the measured soil physicochemical properties. Across the complete dataset, both bacterial and fungal community composition were associated with flavonoid variation, and CT was characterized by a predicted functional profile related to carbohydrate conversion and redox metabolism. Rhizosphere microorganisms and their metabolites can participate in plant–microbe signaling and alter the chemical environment surrounding roots (Korenblum et al., 2022). However, these dataset-level associations cannot demonstrate that microbial changes mediated the CT flavonoid response. Plant transcriptomic, enzyme-activity, and metabolomic measurements would be required to determine whether the phenylpropanoid and flavonoid biosynthetic pathways were activated in tea leaves (Liu et al., 2021).

In CJ, increases in AP and AK and the highest mean fungal Shannon index were accompanied by lower tea-leaf flavonoid content. Mushroom cultivation substrates and spent substrate can support microbial decomposition and nutrient release (Phan and Sabaratnam, 2012), and tea–*Pleurotus ostreatus* intercropping has previously been shown to alter soil nutrient conditions and microbial community structure (Yang et al., 2024). Nevertheless, the present results indicate that greater nutrient availability and fungal diversity do not necessarily translate into a uniform improvement in tea biochemical quality. The coexistence of higher AP and AK with lower flavonoid content may reflect nutrient imbalance, altered plant resource allocation, microbial nutrient immobilization, or other unmeasured environmental factors.

In HJ, the higher soil pH represented a less acidic rhizosphere condition and was accompanied by a distinct bacterial community. Soil pH can influence nutrient solubility, elemental uptake, and tea-leaf biochemical composition (Jia et al., 2024). However, HJ did not significantly increase tea polyphenol content and had significantly lower soluble sugar and flavonoid contents than HJC. These contrasting responses indicate that alleviation of soil acidity did not result in uniform improvement across all tea-quality components. Therefore, reduced soil acidity should not be regarded as equivalent to an overall enhancement of tea quality.

**Figure 7:**
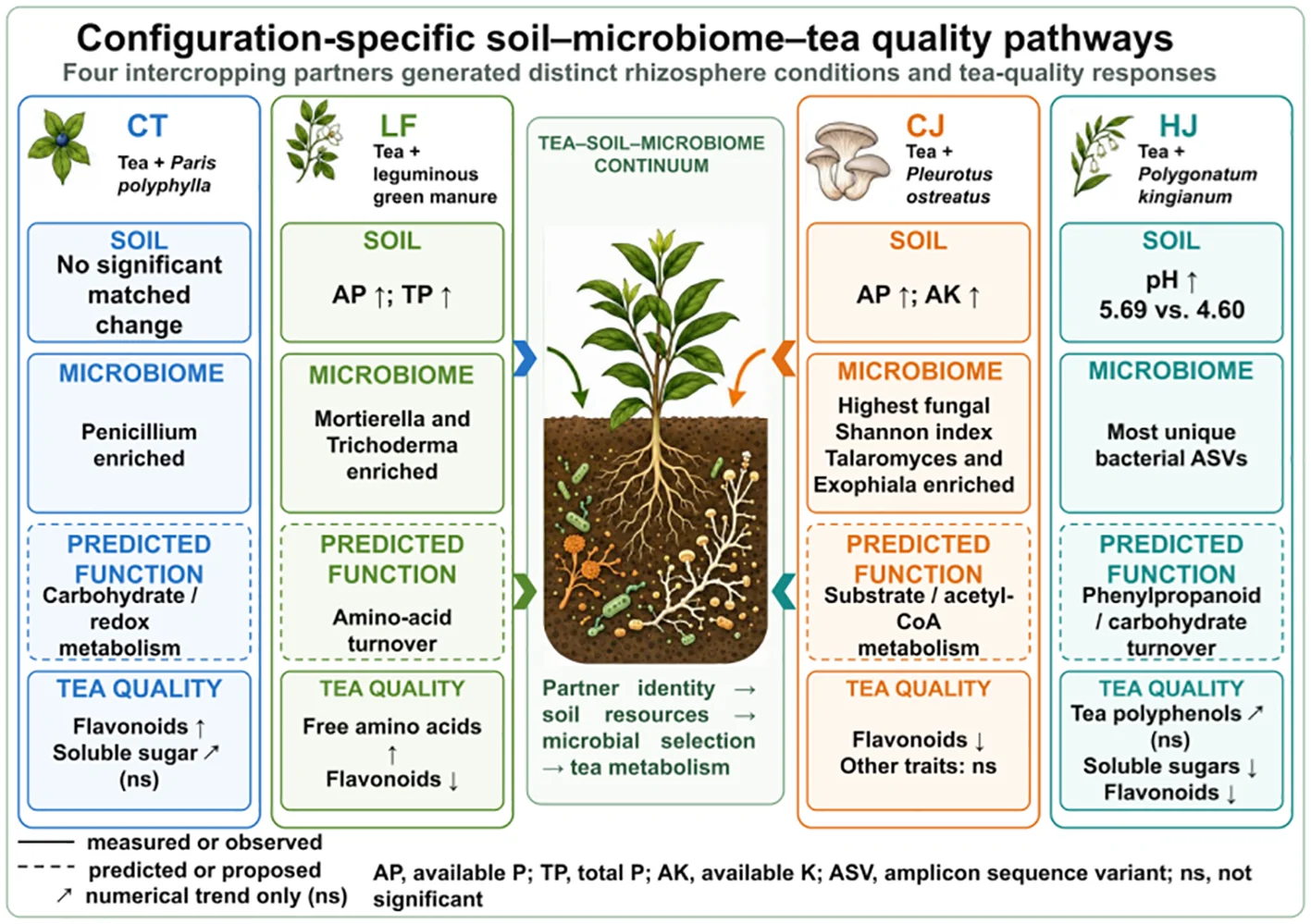
Proposed soil–microbiome–tea quality pathways under different intercropping systems. Solid boxes represent measured results, whereas dashed boxes indicate predicted functions or proposed pathways. Arrows indicate changes relative to the corresponding monoculture controls; ↗ indicates a non-significant increasing trend. CT, Paris polyphylla–tea; LF, leguminous green manure–tea; CJ, *Pleurotus ostreatus*–tea; HJ, *Polygonatum kingianum*–tea. AP, available phosphorus; TP, total phosphorus; AK, available potassium; ASV, amplicon sequence variant; ns, not significant.

Collectively, the integrated evidence supports a configuration-dependent working model in which intercropping-partner identity modifies soil resources and rhizosphere conditions and may consequently select different microbial assemblages and predicted functional profiles. These changes were accompanied by distinct tea biochemical profiles that may reflect differences in plant carbon–nitrogen allocation (**Figure 7**). However, because the present study is based on field associations and marker-gene-based functional inference, the dashed links in the proposed model represent testable hypotheses rather than confirmed causal pathways (Djemiel et al., 2022; Douglas et al., 2020).

### 4.5 Agronomic implications and limitations

The four intercropping configurations may therefore be suited to different management objectives. LF may be useful when increasing free amino acid content and improving soil P status are priorities, although its reduction in flavonoids should also be considered. CT may be selected for increasing flavonoid content, but the increase in soluble sugar was not statistically significant, and its soil effects were limited. CJ increased AP and AK but reduced flavonoids and exhibited substantial variability in AK. HJ was the most effective system for increasing soil pH and showed the largest numerical increase in tea polyphenols; however, soluble sugar and flavonoid contents were significantly lower than those in HJC. Consequently, no single configuration can be considered superior for all soil and tea-quality indicators.

The proposed pathways require validation because root exudates, soil organic-N fractions, microbial genes, enzyme activities, and plant metabolic fluxes were not measured directly. Future experiments should incorporate larger numbers of independently replicated plots, multiple sampling seasons, root-exudate characterization, targeted soil N and P fractions, shotgun metagenomics or metatranscriptomics, and tea-root and leaf transcriptomic or metabolomic analyses. Measurements of tea yield, sensory quality, management cost, and economic return are also needed before recommending a particular intercropping system for commercial application.

## 5 Conclusions

The four intercropping configurations produced distinct, partner-specific effects on the tea rhizosphere and leaf biochemical quality. LF increased leaf free amino acids and soil AP and TP, whereas CT increased leaf flavonoids and exhibited the highest mean soluble sugar content. CJ increased soil AP and AK but reduced leaf flavonoids. HJ significantly increased soil pH and showed the largest numerical increase in tea polyphenols, although it also reduced soluble sugar and flavonoid contents. The four configurations were associated with distinct microbial assemblages and predicted functional potentials, supporting configuration-specific links among soil conditions, rhizosphere microorganisms, and tea biochemical traits. However, these inferred pathways require direct experimental verification. Therefore, intercropping partners should be selected according to specific soil-management and tea-quality objectives rather than assuming that a single configuration can improve all measured properties.

## Data Availability

The original contributions presented in the study are included in the article/**Supplementary Material**. Further inquiries can be directed to the corresponding authors.
