# Supplementary material for: Effects of different intercropping patterns on tea rhizosphere environment and leaf quality
Source: Front Plant Sci. 2026 Aug 11;17:1914455. doi: 10.3389/fpls.2026.1914455 (PMC13503516; doi:10.3389/fpls.2026.1914455)
Supplement: Supplementary file 1 [file Table1.docx]

Supplementary Material

# 1 Supplementary Figures and Tables

**Table S1. Quality components of tea leaves.**

| Treatment | Amino acid (%) | Tea polyphenol (%) | Flavonoids (%) | Soluble sugar (%) |
| --- | --- | --- | --- | --- |
| CT | 1.76 ± 0.08ab | 17.32 ± 0.70b | 10.43 ± 0.17a | 11.77 ± 0.57a |
| CTC | 1.75 ± 0.24ab | 17.41 ± 0.35b | 8.62 ± 0.24c | 10.71 ± 0.53a |
| LF | 2.20 ± 0.13a | 21.12 ± 1.49a | 4.81 ± 0.08f | 8.48 ± 0.61bc |
| LFC | 1.72 ± 0.07b | 19.87 ± 0.95a | 6.53 ± 0.13e | 8.70 ± 0.11bc |
| CJ | 1.75 ± 0.25ab | 20.41 ± 0.85a | 4.57 ± 0.16f | 7.60 ± 0.20c |
| CJC | 1.67 ± 0.09b | 20.34 ± 0.19a | 9.18 ± 0.15b | 8.12 ± 0.22bc |
| HJ | 1.64 ± 0.17b | 19.02 ± 0.60ab | 7.08 ± 0.08d | 9.08 ± 0.34b |
| HJC | 1.79 ± 0.21ab | 17.30 ± 0.37b | 8.59 ± 0.13c | 10.61 ± 0.98a |

Note: Values are presented as the mean ± standard deviation (n = 3). Means within the same column that do not share a common lowercase letter are significantly different according to Tukey’s HSD test following two-way ANOVA (P < 0.05).

**Table S2. Two-way ANOVA results for tea-leaf biochemical properties**

| Parameter | Source of variation | df | Sum of squares | Mean square | F | P | Partial η² |
| --- | --- | --- | --- | --- | --- | --- | --- |
| Amino acid content (%) | Intercropping configuration | 3 | 0.2537 | 0.0846 | 2.9141 | 0.066 | 0.353 |
| Amino acid content (%) | Cropping system | 1 | 0.0666 | 0.0666 | 2.2945 | 0.149 | 0.125 |
| Amino acid content (%) | Configuration × system | 3 | 0.3300 | 0.1100 | 3.7912 | 0.031 | 0.415 |
| Amino acid content (%) | Error | 16 | 0.4643 | 0.0290 |  |  |  |
| Amino acid content (%) | Corrected total | 23 | 1.1146 |  |  |  |  |
| Tea polyphenol content (%) | Intercropping configuration | 3 | 44.7195 | 14.9065 | 23.9506 | <0.001 | 0.818 |
| Tea polyphenol content (%) | Cropping system | 1 | 3.2807 | 3.2807 | 5.2712 | 0.036 | 0.248 |
| Tea polyphenol content (%) | Configuration × system | 3 | 3.5610 | 1.1870 | 1.9072 | 0.169 | 0.263 |
| Tea polyphenol content (%) | Error | 16 | 9.9581 | 0.6224 |  |  |  |
| Tea polyphenol content (%) | Corrected total | 23 | 61.5193 |  |  |  |  |
| Flavonoid content (%) | Intercropping configuration | 3 | 47.7407 | 15.9136 | 700.0928 | <0.001 | 0.992 |
| Flavonoid content (%) | Cropping system | 1 | 13.6263 | 13.6263 | 599.4674 | <0.001 | 0.974 |
| Flavonoid content (%) | Configuration × system | 3 | 31.1137 | 10.3712 | 456.2664 | <0.001 | 0.988 |
| Flavonoid content (%) | Error | 16 | 0.3637 | 0.0227 |  |  |  |
| Flavonoid content (%) | Corrected total | 23 | 92.8444 |  |  |  |  |
| Soluble sugar content (%) | Intercropping configuration | 3 | 39.6590 | 13.2197 | 48.8149 | <0.001 | 0.902 |
| Soluble sugar content (%) | Cropping system | 1 | 0.5509 | 0.5509 | 2.0343 | 0.173 | 0.113 |
| Soluble sugar content (%) | Configuration × system | 3 | 5.1027 | 1.7009 | 6.2807 | 0.005 | 0.541 |
| Soluble sugar content (%) | Error | 16 | 4.3330 | 0.2708 |  |  |  |
| Soluble sugar content (%) | Corrected total | 23 | 49.6456 |  |  |  |  |

Note. Intercropping configuration (CT, LF, CJ, and HJ) and cropping system (intercropping versus corresponding monoculture) were treated as fixed factors in a balanced 4 × 2 design (n = 3 per cell; N = 24). Partial η² = SSeffect/(SSeffect + SSerror). Residual Shapiro–Wilk tests yielded P = 0.760–0.988, and median-centered Levene tests yielded P = 0.653–0.916; therefore, no transformation was required.

**Table S3. Two-way ANOVA of soil physicochemical properties**

| Variable | Source of variation | df | Sum of squares | Mean square | F | P |
| --- | --- | --- | --- | --- | --- | --- |
| pH | Intercropping configuration | 3 | 1.814 | 0.6046 | 12.223 | < 0.001 |
|  | Cropping system | 1 | 0.5689 | 0.5689 | 11.500 | 0.004 |
|  | Configuration × system | 3 | 1.759 | 0.5865 | 11.856 | < 0.001 |
|  | Residual | 16 | 0.7915 | 0.0495 | — | — |
| SOM | Intercropping configuration | 3 | 10,347.85 | 3,449.28 | 43.443 | < 0.001 |
|  | Cropping system | 1 | 176.75 | 176.75 | 2.226 | 0.155 |
|  | Configuration × system | 3 | 8.011 | 2.670 | 0.034 | 0.991 |
|  | Residual | 16 | 1,270.38 | 79.399 | — | — |
| TN | Intercropping configuration | 3 | 0.0695 | 0.0232 | 20.589 | < 0.001 |
|  | Cropping system | 1 | 0.00186 | 0.00186 | 1.657 | 0.216 |
|  | Configuration × system | 3 | 0.00161 | 0.00054 | 0.478 | 0.702 |
|  | Residual | 16 | 0.0180 | 0.00113 | — | — |
| AP | Intercropping configuration | 3 | 2.379 | 0.7929 | 141.556 | < 0.001 |
|  | Cropping system | 1 | 0.6179 | 0.6179 | 110.324 | < 0.001 |
|  | Configuration × system | 3 | 1.165 | 0.3882 | 69.306 | < 0.001 |
|  | Residual | 16 | 0.0896 | 0.00560 | — | — |
| AK | Intercropping configuration | 3 | 0.1641 | 0.0547 | 1.683 | 0.211 |
|  | Cropping system | 1 | 0.3350 | 0.3350 | 10.304 | 0.005 |
|  | Configuration × system | 3 | 0.2698 | 0.0899 | 2.766 | 0.076 |
|  | Residual | 16 | 0.5202 | 0.0325 | — | — |
| TK | Intercropping configuration | 3 | 854.12 | 284.71 | 346.291 | < 0.001 |
|  | Cropping system | 1 | 3.489 | 3.489 | 4.244 | 0.056 |
|  | Configuration × system | 3 | 5.330 | 1.777 | 2.161 | 0.133 |
|  | Residual | 16 | 13.155 | 0.8222 | — | — |
| TP | Intercropping configuration | 3 | 1.412 | 0.4706 | 6.800 | 0.004 |
|  | Cropping system | 1 | 2.695 | 2.695 | 38.943 | < 0.001 |
|  | Configuration × system | 3 | 2.305 | 0.7682 | 11.101 | < 0.001 |
|  | Residual | 16 | 1.107 | 0.0692 | — | — |
| CEC | Intercropping configuration | 3 | 257.62 | 85.874 | 41.048 | < 0.001 |
|  | Cropping system | 1 | 21.599 | 21.599 | 10.324 | 0.005 |
|  | Configuration × system | 3 | 0.5985 | 0.1995 | 0.095 | 0.961 |
|  | Residual | 16 | 33.472 | 2.092 | — | — |

**Note:** The fixed factors were intercropping configuration (CT, LF, CJ, and HJ) and cropping system (intercropping vs. monoculture). Each treatment combination included three independent replicates. ᵃAP, AK, and TP were analyzed after Box–Cox transformation (λ = −0.461, −0.149, and −2.188, respectively). Residual normality and homogeneity of variance were confirmed by Shapiro–Wilk and median-centered Levene tests (all P > 0.05 after transformation where required).

**Table S4. Alpha-diversity indices of rhizosphere fungal and bacterial communities under different intercropping systems**

| Community | Estimator | CT | CTC | LF | LFC | CJ | CJC | HJ | HJC | Hstatistic | P-value |
| --- | --- | --- | --- | --- | --- | --- | --- | --- | --- | --- | --- |
| Fungi | Sobs | 600.67 ± 113.60 | 680.00 ± 121.45 | 907.67 ± 204.18 | 614.33 ± 117.33 | 751.67 ± 79.58 | 597.67 ± 38.94 | 813.33 ± 92.99 | 611.33 ± 208.87 | 10.7867 | 0.14820 |
| Fungi | ACE | 606.21 ± 116.34 | 691.75 ± 129.91 | 924.14 ± 217.33 | 624.37 ± 129.69 | 754.81 ± 80.22 | 599.04 ± 38.77 | 820.97 ± 102.71 | 621.10 ± 214.93 | 10.5467 | 0.15970 |
| Fungi | Chao | 604.69 ± 115.95 | 689.31 ± 124.80 | 919.75 ± 213.95 | 622.42 ± 127.32 | 753.25 ± 79.66 | 598.21 ± 38.68 | 819.65 ± 101.82 | 618.82 ± 213.76 | 10.5467 | 0.15970 |
| Fungi | Shannon | 4.2318 ± 0.1851 | 4.2025 ± 0.1074 | 4.8307 ± 0.0653 | 3.9749 ± 0.8304 | 5.0509 ± 0.0356 | 4.7566 ± 0.1444 | 4.8164 ± 0.2004 | 4.3140 ± 0.3427 | 18.2400 | 0.01093 |
| Fungi | Simpson | 0.05307 ± 0.00321 | 0.05451 ± 0.00919 | 0.02757 ± 0.00651 | 0.10324 ± 0.12694 | 0.01612 ± 0.00064 | 0.02368 ± 0.00212 | 0.02639 ± 0.01029 | 0.04034 ± 0.01338 | 17.3733 | 0.01514 |
| Fungi | Coverage | 0.99974 ± 0.00013 | 0.99953 ± 0.00027 | 0.99936 ± 0.00046 | 0.99963 ± 0.00042 | 0.99982 ± 0.00004 | 0.99992 ± 0.00002 | 0.99966 ± 0.00037 | 0.99961 ± 0.00029 | 7.1357 | 0.41490 |
| Bacteria | Sobs | 2553.00 ± 298.02 | 2043.33 ± 525.51 | 2944.33 ± 400.17 | 2795.67 ± 256.09 | 3039.33 ± 270.02 | 2452.33 ± 182.89 | 3184.67 ± 230.57 | 2034.67 ± 620.93 | 15.1600 | 0.03400 |
| Bacteria | ACE | 2592.28 ± 327.32 | 2059.21 ± 524.53 | 3004.29 ± 452.93 | 2835.76 ± 268.44 | 3089.56 ± 319.94 | 2470.58 ± 196.80 | 3238.69 ± 279.32 | 2066.97 ± 642.41 | 14.2133 | 0.04751 |
| Bacteria | Chao | 2568.27 ± 310.28 | 2046.94 ± 524.60 | 2961.65 ± 420.30 | 2805.37 ± 258.47 | 3054.38 ± 286.89 | 2456.05 ± 187.43 | 3199.71 ± 248.46 | 2045.56 ± 629.08 | 14.3467 | 0.04535 |
| Bacteria | Shannon | 7.1807 ± 0.1057 | 6.7474 ± 0.4499 | 7.2540 ± 0.1003 | 7.2318 ± 0.0964 | 7.3027 ± 0.0882 | 7.0513 ± 0.1634 | 7.3992 ± 0.1261 | 6.7403 ± 0.4186 | 15.5867 | 0.02917 |
| Bacteria | Simpson | 0.00142 ± 0.00018 | 0.00349 ± 0.00268 | 0.00143 ± 0.00025 | 0.00135 ± 0.00019 | 0.00140 ± 0.00012 | 0.00210 ± 0.00071 | 0.00127 ± 0.00040 | 0.00279 ± 0.00121 | 12.1333 | 0.09626 |
| Bacteria | Coverage | 0.99724 ± 0.00190 | 0.99879 ± 0.00038 | 0.99636 ± 0.00310 | 0.99728 ± 0.00122 | 0.99688 ± 0.00301 | 0.99864 ± 0.00100 | 0.99655 ± 0.00257 | 0.99775 ± 0.00143 | 4.9855 | 0.66170 |

**Table S5. Predicted abundances and log₂ fold changes of upregulated enzyme functions in rhizosphere bacterial and fungal communities under four intercropping systems.**

**A. Bacterial community (16S rRNA)**

| Comparison | EC no. | Predicted enzyme function | Intercropping abundance | Control abundance | log₂FC |
| --- | --- | --- | --- | --- | --- |
| CT vs CTC | 3.1.22.1 | Deoxyribonuclease II | 27.67 | 2.43 | 3.51 |
| CT vs CTC | 1.5.1.10 | Saccharopine dehydrogenase | 225.44 | 23.00 | 3.29 |
| CT vs CTC | 3.4.24.56 | Insulysin | 85.00 | 12.33 | 2.78 |
| CT vs CTC | 2.4.1.284 | 2-deoxystreptamine glucosyltransferase | 12.22 | 1.79 | 2.77 |
| CT vs CTC | 2.4.1.283 | 2-deoxystreptamine N-acetyl-D-glucosaminyltransferase | 12.22 | 1.79 | 2.77 |
| CT vs CTC | 1.1.1.82 | Malate dehydrogenase (NADP(+)) | 72.33 | 10.67 | 2.76 |
| CT vs CTC | 3.2.1.68 | Isoamylase | 72.33 | 10.67 | 2.76 |
| CT vs CTC | 2.5.1.2 | Thiamine pyridinylase | 46.95 | 7.42 | 2.66 |
| CT vs CTC | 5.4.99.7 | Lanosterol synthase | 28.00 | 5.01 | 2.48 |
| CT vs CTC | 2.1.1.178 | 16S rRNA (cytosine(1407)-C(5))-methyltransferase | 12.14 | 2.43 | 2.32 |
| LF vs LFC | 2.2.1.10 | 2-amino-3,7-dideoxy-D-threo-hept-6-ulosonate synthase | 15.48 | 1.01 | 3.94 |
| LF vs LFC | 2.3.1.169 | CO-methylating acetyl-CoA synthase | 116.50 | 45.67 | 1.35 |
| LF vs LFC | 2.1.1.258 | Co-methyltransferase | 124.33 | 52.83 | 1.23 |
| LF vs LFC | 3.4.21.116 | SpoIVB peptidase | 581.10 | 287.49 | 1.02 |
| LF vs LFC | 3.4.24.78 | GPR endopeptidase | 580.43 | 287.49 | 1.01 |
| LF vs LFC | 2.7.1.66 | Undecaprenol kinase | 1,781.47 | 1,125.40 | 0.66 |
| LF vs LFC | 1.3.99.8 | 2-furoyl-CoA dehydrogenase | 1,933.73 | 1,365.77 | 0.50 |
| LF vs LFC | 1.15.1.2 | Superoxide reductase | 235.25 | 172.06 | 0.45 |
| LF vs LFC | 4.3.1.1 | Aspartate ammonia-lyase | 7,879.80 | 6,334.30 | 0.31 |
| CJ vs CJC | 5.3.99.7 | Styrene-oxide isomerase | 36.00 | 0.01 | 11.43 |
| CJ vs CJC | 1.1.1.373 | Sulfolactaldehyde 3-reductase | 31.73 | 1.34 | 4.56 |
| CJ vs CJC | 1.21.4.2 | Glycine reductase | 26.27 | 2.01 | 3.71 |
| CJ vs CJC | 2.4.1.173 | Sterol 3-beta-glucosyltransferase | 41.39 | 4.34 | 3.25 |
| CJ vs CJC | 3.2.1.6 | Endo-1,3(4)-beta-glucanase | 16.56 | 1.89 | 3.13 |
| CJ vs CJC | 3.4.11.15 | Aminopeptidase Y | 96.42 | 21.92 | 2.14 |
| CJ vs CJC | 2.3.3.8 | ATP citrate synthase | 194.00 | 50.00 | 1.96 |
| CJ vs CJC | 1.10.3.3 | L-ascorbate oxidase | 35.17 | 10.17 | 1.79 |
| CJ vs CJC | 1.14.21.9 | Mycocyclosin synthase | 10.96 | 3.69 | 1.57 |
| CJ vs CJC | 1.3.99.23 | All-trans-retinol 13,14-reductase | 439.56 | 162.44 | 1.44 |
| HJ vs HJC | 5.3.99.2 | Prostaglandin-D synthase | 28.00 | 0.01 | 11.07 |
| HJ vs HJC | 2.6.1.64 | Glutamine--phenylpyruvate transaminase | 17.67 | 1.34 | 3.72 |
| HJ vs HJC | 4.4.1.13 | Cysteine-S-conjugate beta-lyase | 17.67 | 1.34 | 3.72 |
| HJ vs HJC | 2.6.1.7 | Kynurenine--oxoglutarate transaminase | 17.67 | 1.34 | 3.72 |
| HJ vs HJC | 1.14.19.20 | Delta(7)-sterol 5(6)-desaturase | 95.00 | 7.34 | 3.69 |
| HJ vs HJC | 5.1.3.8 | N-acylglucosamine 2-epimerase | 143.89 | 12.24 | 3.56 |
| HJ vs HJC | 2.4.2.6 | Nucleoside deoxyribosyltransferase | 28.41 | 2.79 | 3.35 |
| HJ vs HJC | 5.3.1.31 | Sulfoquinovose isomerase | 133.32 | 15.00 | 3.15 |
| HJ vs HJC | 1.1.1.105 | All-trans-retinol dehydrogenase (NAD(+)) | 133.00 | 15.00 | 3.15 |
| HJ vs HJC | 2.7.7.74 | 1L-myo-inositol 1-phosphate cytidylyltransferase | 30.00 | 4.00 | 2.91 |

**B. Fungal community (ITS)**

| Comparison | EC no. | Predicted enzyme function | Intercropping abundance | Control abundance | log₂FC |
| --- | --- | --- | --- | --- | --- |
| CT vs CTC | 1.1.1.10 | L-xylulose reductase | 25.00 | 0.03 | 9.56 |
| CT vs CTC | 4.2.1.76 | UDP-glucose 4,6-dehydratase | 25.00 | 0.03 | 9.56 |
| CT vs CTC | 1.1.1.153 | Sepiapterin reductase | 25.00 | 0.03 | 9.56 |
| CT vs CTC | 3.6.3.31 | Polyamine-transporting ATPase | 25.00 | 0.03 | 9.56 |
| CT vs CTC | 2.5.1.43 | Nicotianamine synthase | 8,975.93 | 2,294.65 | 1.97 |
| CT vs CTC | 1.13.11.33 | Arachidonate 15-lipoxygenase | 7,513.58 | 2,158.99 | 1.80 |
| CT vs CTC | 4.3.1.15 | Diaminopropionate ammonia-lyase | 1,018.00 | 342.99 | 1.57 |
| CT vs CTC | 5.3.99.2 | Prostaglandin-D synthase | 1,018.00 | 342.99 | 1.57 |
| CT vs CTC | 5.1.99.3 | Allantoin racemase | 1,018.00 | 342.99 | 1.57 |
| CT vs CTC | 3.2.1.17 | Lysozyme | 1,018.00 | 342.99 | 1.57 |
| LF vs LFC | 1.2.1.44 | Cinnamoyl-CoA reductase | 387.00 | 0.03 | 13.52 |
| LF vs LFC | 3.6.3.31 | Polyamine-transporting ATPase | 387.00 | 0.03 | 13.52 |
| LF vs LFC | 3.4.21.69 | Protein C (activated) | 387.00 | 0.03 | 13.52 |
| LF vs LFC | 2.7.1.95 | Kanamycin kinase | 387.00 | 0.03 | 13.52 |
| LF vs LFC | 3.1.3.29 | N-acylneuraminate-9-phosphatase | 387.00 | 0.03 | 13.52 |
| LF vs LFC | 1.1.1.153 | Sepiapterin reductase | 1,081.00 | 2.34 | 8.85 |
| LF vs LFC | 3.6.3.10 | Hydrogen/potassium-exchanging ATPase | 11.34 | 0.03 | 8.42 |
| LF vs LFC | 4.3.1.3 | Histidine ammonia-lyase | 11.34 | 0.03 | 8.42 |
| LF vs LFC | 2.4.1.50 | Procollagen galactosyltransferase | 821.00 | 5.00 | 7.36 |
| LF vs LFC | 2.7.4.10 | Nucleoside-triphosphate--adenylate kinase | 701.00 | 10.01 | 6.13 |
| CJ vs CJC | 3.1.3.29 | N-acylneuraminate-9-phosphatase | 22.00 | 0.03 | 9.38 |
| CJ vs CJC | 3.4.21.69 | Protein C (activated) | 22.00 | 0.03 | 9.38 |
| CJ vs CJC | 2.7.1.95 | Kanamycin kinase | 22.00 | 0.03 | 9.38 |
| CJ vs CJC | 1.2.1.44 | Cinnamoyl-CoA reductase | 22.00 | 0.83 | 4.73 |
| CJ vs CJC | 3.1.6.2 | Steryl-sulfatase | 190.32 | 19.34 | 3.30 |
| CJ vs CJC | 1.1.1.219 | Dihydrokaempferol 4-reductase | 3,822.33 | 527.34 | 2.86 |
| CJ vs CJC | 4.1.1.29 | Sulfinoalanine decarboxylase | 3,822.33 | 527.34 | 2.86 |
| CJ vs CJC | 3.4.21.62 | Subtilisin | 3,822.33 | 527.34 | 2.86 |
| CJ vs CJC | 4.1.99.1 | Tryptophanase | 1,591.67 | 372.67 | 2.09 |
| CJ vs CJC | 3.1.3.67 | Phosphatidylinositol-3,4,5-trisphosphate 3-phosphatase | 2,370.00 | 591.36 | 2.00 |
| HJ vs HJC | 2.4.1.50 | Procollagen galactosyltransferase | 20.66 | 0.03 | 9.29 |
| HJ vs HJC | 1.13.11.34 | Arachidonate 5-lipoxygenase | 17.00 | 0.33 | 5.69 |
| HJ vs HJC | 1.14.13.11 | Trans-cinnamate 4-monooxygenase | 129.99 | 19.00 | 2.77 |
| HJ vs HJC | 2.4.1.135 | Galactosylgalactosylxylosylprotein 3-beta-glucuronosyltransferase | 114.66 | 17.00 | 2.75 |
| HJ vs HJC | 3.2.1.10 | Oligo-1,6-glucosidase | 105.00 | 16.20 | 2.70 |
| HJ vs HJC | 1.3.1.45 | 2'-hydroxyisoflavone reductase | 2,001.73 | 355.53 | 2.49 |
| HJ vs HJC | 2.7.3.3 | Arginine kinase | 47.68 | 9.33 | 2.35 |
| HJ vs HJC | 3.6.3.10 | Hydrogen/potassium-exchanging ATPase | 46.35 | 9.33 | 2.31 |
| HJ vs HJC | 4.3.1.3 | Histidine ammonia-lyase | 46.35 | 9.33 | 2.31 |
| HJ vs HJC | 1.14.13.39 | Nitric-oxide synthase (NADPH) | 427.99 | 90.32 | 2.24 |

**Note.** Values are predicted enzyme abundances. log₂FC values were retained as supplied and represent enrichment in each intercropping treatment relative to its corresponding monoculture tea control. Bacterial and fungal functions were inferred from 16S rRNA and ITS data, respectively, using PICRUSt2. Up to 10 enzyme functions are listed per comparison.

**Abbreviations:** CT, *Paris polyphylla*–tea; LF, *Vicia villosa* var. (hairy vetch)–tea; CJ, *Pleurotus ostreatus*–tea; HJ, *Polygonatum kingianum*–tea. The suffix C denotes the corresponding monoculture tea control.
